# G protein‐coupled receptor 37‐like 1 modulates astrocyte glutamate transporters and neuronal NMDA receptors and is neuroprotective in ischemia

**DOI:** 10.1002/glia.23198

**Published:** 2017-08-10

**Authors:** Sarah Jolly, Narges Bazargani, Alejandra C. Quiroga, Nigel P. Pringle, David Attwell, William D. Richardson, Huiliang Li

**Affiliations:** ^1^ Wolfson Institute for Biomedical Research, University College London London WC1E 6BT United Kingdom; ^2^ Department of Neuroscience, Physiology and Pharmacology University College London London WC1E 6BT United Kingdom

**Keywords:** *Gpr37l1‐GFP* mice, *Gpr37l1* knockout mice, MCAO, neuroprotection, prosaposin

## Abstract

We show that the G protein‐coupled receptor GPR37‐like 1 (GPR37L1) is expressed in most astrocytes and some oligodendrocyte precursors in the mouse central nervous system. This contrasts with GPR37, which is mainly in mature oligodendrocytes. Comparison of wild type and *Gpr37l1^–/–^* mice showed that loss of GPR37L1 did not affect the input resistance or resting potential of astrocytes or neurons in the hippocampus. However, GPR37L1‐mediated signalling inhibited astrocyte glutamate transporters and – surprisingly, given its lack of expression in neurons – reduced neuronal NMDA receptor (NMDAR) activity during prolonged activation of the receptors as occurs in ischemia. This effect on NMDAR signalling was not mediated by a change in the release of D‐serine or TNF‐α, two astrocyte‐derived agents known to modulate NMDAR function. After middle cerebral artery occlusion, *Gpr37l1* expression was increased around the lesion. Neuronal death was increased by ∼40% in *Gpr37l1^–/–^* brain compared to wild type in an in vitro model of ischemia. Thus, GPR37L1 protects neurons during ischemia, presumably by modulating extracellular glutamate concentration and NMDAR activation.

## 1 INTRODUCTION

Activation of receptors on astrocytes is increasingly thought to modulate the activity and function of neurons. Release of astrocyte‐derived “gliotransmitters” such as glutamate and D‐serine, triggered by activation of receptors on astrocytes by signals from neurons or other cells, can alter synaptic transmitter release and the excitability of neurons (Bazargani and Attwell, 2016). However, the functions of most glial receptors are poorly understood. Here, we examine the function of one such glial‐restricted receptor, the G protein‐coupled receptor GPR37‐like 1 (GPR37L1).

We identified GPR37L1 as a potential astrocyte‐specific receptor during a visual screen of the Allen Brain Atlas gene expression database (N.P.P. unpublished). GPR37L1 belongs to the Class A rhodopsin‐like receptor subfamily of GPCRs. The *Gpr37l1* coding sequence was first identified by sequence similarity to the endothelin type B receptor gene, but GPR37L1 is unable to bind endothelin or related peptides (Leng, Gu, Simerly, & Spindel, 1999; Valdenaire et al., 1998). It is highly expressed in the central nervous system (CNS), heart and gastrointestinal tract (Freeman, 2010; Ito et al., 2009; Min et al., 2010; Valdenaire et al., 1998). GPR37L1 shares >40% amino acid sequence similarity with its close relative GPR37, which is also expressed in the CNS. Transcriptome studies suggest that *Gpr37l1* is expressed mainly in astrocytes, oligodendrocyte precursors (OPs) and newly formed oligodendrocytes (OLs) in humans and mice (web.stanford.edu/group/barres_lab/cgi‐bin/geneSearch.py?geneNameIn = gpr37l1), while *Gpr37* is mainly in newly formed and myelinating OLs (web.stanford.edu/group/barres_lab/cgi‐bin/geneSearch.py?geneNameIn = gpr37) (Imai et al., 2001; Zhang et al., 2014). GPR37 is a substrate of Parkin, an E3 ubiquitin ligase that might regulate the dopaminergic system (Imai et al., 2001). It also regulates OL differentiation and myelination (Yang, Vainshtein, Maik‐Rachline, & Peles, 2016). In contrast, little is known about the function of GPR37L1 in the CNS, except that it might modulate development of the cerebellum by regulating sonic hedgehog signalling (Marazziti et al., 2013). Recently, the polypeptide “prosaposin” (also known as PSAP) was identified as a potential ligand for both GPR37 and GPR37L1 (Meyer, Giddens, Schaefer, & Hall, 2013). Prosaposin can be secreted into the extracellular space and this is enhanced following conditions of cellular stress such as ischemia (Costain et al., 2010; Hiraiwa et al., 2003; Yokota, Uchijima, Nishizawa, Namba, & Koide, 2001). Prosaposin and prosaptide (an active fragment of prosaposin) have neuroprotective and glioprotective properties (Meyer, Giddens, Coleman, & Hall, 2014; Morita et al., 2001; Sano et al., 1994) by acting on GPR37 and GPR37L1. However, a separate study has suggested that GPR37L1 is constitutively active and that its activity is regulated by proteolytic cleavage near the N‐terminus (Coleman et al., 2016). It is therefore unclear whether GPR37L1 activation is triggered by binding of an extracellular ligand (like prosaposin) or by post‐translational modification or cleavage.

We report that GPR37 and GPR37L1 are expressed in the postnatal CNS in non‐overlapping cell populations. While GPR37 is expressed mainly in differentiated OLs, GPR37L1 is expressed in astrocytes and OPs. We found that (1) GPR37L1 expression does not change the basic membrane properties of hippocampal astrocytes or neurons, (2) GPR37L1 mRNA expression is upregulated in ischemia in vivo, (3) GPR37L1 expression and signalling activated by its ligand prosaptide are neuroprotective in ischemic brain slices, and (4) prosaptide‐evoked GPR37L1‐signalling inhibits glutamate transporters in astrocytes and reduces neuronal NMDAR activity. We suggest that the latter two effects combine to confer neuroprotection during ischemia.

## 2 MATERIALS AND METHODS

### 2.1 Mice

Mouse husbandry and experimentation conformed with UK Home Office regulations, UCL Ethics Committee guidelines and the UK Animals (Scientific Procedures) Act 1986 and its Amendment Regulations (2012).

*Gpr37l1* knock‐out (KO) mice were from the NIH Mutant Mouse Resource and Research Centers (B6;129S5‐*Gpr37l1^tm1Lex^*/Mmucd). They have a *LacZ‐neo^R^* cassette inserted by homologous recombination into the first exon of the *Gpr37l1* gene, causing a deletion and loss of function of the gene. Mice were maintained on a mixed genetic background (C57BL/6, 129S5) and genotyped using PCR

(primers:neo>5‐gpr:5′‐CGTGATATTGCTGAAGAGCTTG;

$$\text{gpr}-\text{wt}5:\;5^{\prime }-\text{CATCTTTAGGTGGGC}\;\text{ATAGAGC};$$

$$\text{gpr}-\text{ko}-3:\;5^{\prime }-\text{CAGATCTTTGCAGACACTGGAG}).$$

*Gpr37l1‐GFP* transgenic mice were generated by inserting a *lox.GFP.STOP.lox‐DTA–frt.Km^r^.frt* cassette immediately downstream of the *Gpr37l1* initiation codon in a bacterial artificial chromosome (BAC RP23–287O2, Source Bioscience, Nottingham, UK). *STOP* is a series of four simian virus 40 poly‐A addition sites and *DTA* is the diphtheria toxin A‐chain coding sequence. The *frt.Km^r^.frt* element was removed by expressing Flp recombinase in the bacterial host. The modified BAC was linearized with NotI before gel purification and pronuclear injection into C57BL/6, CBA F1 generation oocytes. Genotyping was by PCR using the following primers:

$$\begin{array}{cc} \text{gprF}:5\prime ‐\text{GTTGCAGTGATTGGAGCAGGTC} & \text{gfpR}:5\prime ‐\text{ACTTGAAGAAGTCGTGCTGCT.} \end{array}$$

### 2.2 Immunohistochemistry

Anesthetized mice were transcardially perfused with 0.1 M phosphate buffer (20 mL), pH 7.4 (PBS) followed by 4% (w/v) paraformaldehyde (PFA; Sigma, 50 mL) in PBS. Brains were post‐fixed in 4% PFA overnight at 4°C, cryoprotected in 20% (w/v) sucrose (Sigma), kept overnight at 4°C and frozen in Tissue‐Tek O.C.T. (Sakura Finetek). Coronal brain cryo‐sections (25 µm thick) were blocked with PBS containing 10% (v/v) fetal bovine serum (FBS) and 0.1% (v/v) Triton‐X100 for 1 hr at 20–25°C, and then incubated with primary antibody (Table 1) in PBS with 5% FBS and 0.1% Triton‐X100 at 4°C overnight. The next day, sections were incubated with secondary antibodies (Alexa Fluor‐488, −568 or −647; Thermo Fisher) for 2 hr at 20–25°C. Nuclei were counterstained with Hoechst 33258 (Sigma) and sections were mounted in DAKO fluorescence medium. Anti‐PDGFRA antibody detection employed a goat anti‐rabbit antibody conjugated to biotin (1:200, Jackson ImmunoResearch). For signal amplification, the Vectastain ABC kit (Vector) with Fluorescein (1:100, Perkin Elmer) was used.

**Table 1:** List of primary antibodies

Target	Host species	Dilution	Company	
GFAP	Mouse	1/500	Sigma	
OLIG2	Rabbit	1/500	Millipore	
SOX10	Guinea pig	1/2000	gift from M. Wegner	
PDGFRA	Rabbit	1/500	Cell Signalling	
β‐galactosidase	Rabbit	1/500	Cappel	
S100β	Mouse	1/500	Sigma	
CC1	Mouse	1/200	Calbiochem	
NEUN	Mouse	1/200	Millipore	
GFP	Rat	1/2000	Nacalai Tesque Inc	
IBA1	Rabbit	1/500	WAKO	
vGLUT2	Guinea pig	1/500	Chemicon	
PSD95	Rabbit	1/500	Abcam	
vGAT	Guinea pig	1/500	Synaptic Systems	

For synaptic staining, mice were perfused with 40 mL of PBS and their brains were removed and post‐fixed by immersion in 4% PFA for 1 hr at 20–25°C. Brain cryo‐sections were blocked with PBS containing 20% FBS. Primary and secondary antibodies were incubated in PBS with 10% FBS and 0.3% Triton‐X100. Stacks of 10 images (*z*‐step = 0.36 µm) were made with a Perkin Elmer spinning disk confocal microscope (63× objective). Numbers of PSD95/vGLUT2 double‐positive puncta and VGAT positive puncta per NEUN^+^ cell were counted using Volocity software (3 slices/mouse, 3 images/slice).

### 2.3 In situ hybridization

Our in situ hybridization (ISH) procedure has been described (Jolly, Fudge, Pringle, Richardson, & Li, 2016; http://www.ucl.ac.uk/~ucbzwdr/In%20Situ%20Protocol.pdf). Briefly, coronal brain slices 15 μm thick were collected on glass slides and incubated with digoxigenin (DIG)‐labelled RNA probes. The DIG signal was visualized with alkaline phosphatase (AP)‐conjugated anti‐DIG Fab fragment and Fast Red fluorescence system (Roche). For double ISH, fluorescein (FITC)‐ and DIG‐labelled probes were applied simultaneously. DIG and FITC were detected on consecutive days with horseradish peroxidase (POD)‐conjugated anti‐DIG and anti‐FITC, blocking POD activity with H_2_O_2_ before adding anti‐FITC. The signal was developed by incubating with TSA Plus Fluorescence kits (fluorescein or tyramide cyanine5) (Perkin Elmer).

The plasmids for riboprobe synthesis were IMAGE clone IRAKp961i05207Q (Source Bioscience) for *Gpr37l1* (linearized with EcoR1, transcribed with SP6 RNA polymerase) and IMAGE clone IMAGp998D0613991Q (Source Bioscience) for *Gpr37* (linearized with Sal1, transcribed with T7 polymerase).

### 2.4 Electrophysiology

Electrophysiological recordings from wild type and knockout mice were performed with the experimenter being blind to the mouse genotype.

### 2.5 Slice Preparation

Hippocampal slices (270 μm thick) were prepared from *Gpr37l1^–/–^* mice and *Gpr37l1^+/+^* littermates at postnatal days 14–16 (P14‐P16). Mice were killed by cervical dislocation, followed by decapitation. The head was immersed in ice‐cold slicing solution containing 87 mM NaCl, 25 mM NaHCO_3_, 25 mM glucose, 75 mM sucrose, 2.5 mM KCl, 1.25 mM NaH_2_PO_4_, 0.5 mM CaCl_2_, 7 mM MgCl_2_, 1 mM kynurenic acid (to block glutamate receptors during slicing), pH 7.2–7.4 (gassed with 95% O_2_/5% CO_2_), osmolarity 330–340 mOsm L^−1^. Hippocampal dissection employed the “magic cut” (Bischofberger, Engel, Li, Geiger, & Jonas, 2006) to make slices (on a Vibratome), which were transferred to a heated chamber at 30°C for 40 minutes, then removed and allowed to reach 20–25°C for 20 min prior to recording.

### 2.6 Whole‐cell patch‐clamp recording

Neurons and astrocytes were selected visually for patch‐clamping, and their identities were confirmed from their morphology after diffusion of Alexa Fluor 488 into neurons, or Alexa Fluor 488/594 into astrocytes. When voltage steps were applied, observing a large voltage‐gated sodium current, or a passive current‐voltage relation of low resistance, confirmed that cells were pyramidal neurons, or astrocytes, respectively. Data for the drug responses presented were sampled at 1 kHz and filtered at ≤500 Hz.

### 2.7 Extracellular solutions

When recording, slices were superfused with artificial cerebrospinal fluid (aCSF, via a gravity‐driven system using 60 mL syringes connected to tubes which merged into a single outlet) containing 140 mM NaCl, 10 mM HEPES, 10 mM glucose, 2.5 mM KCl, 2 mM CaCl_2_, 1 mM NaH_2_PO_4_, 1 mM MgCl_2_, pH 7.4 set with NaOH, osmolarity 300 mOsm L^−1^ (gassed with 100% O_2_). The flow rate was 3–4 mL min^−1^. Electrophysiology experiments were at 20–25°C.

For glutamate receptor currents from CA1 pyramidal neurons, voltage‐clamp recordings were made at −29 mV (including the −14 mV junction potential for internal solution containing K‐gluconate), in order to remove Mg^2+^‐block of NMDARs. Voltage‐clamp recordings from CA1 neurons were performed in tetrodotoxin (TTX, 400 nM, Tocris) to block action potentials and picrotoxin (100 μM, Sigma) to block GABA_A_ receptors. For Figure 6a–c, kainate (3 μM, Sigma) was added to activate AMPA receptors (AMPARs) and kainate receptors (KARs), in D‐AP5 (50 μM, Tocris) to block NMDARs. Experiments measuring responses to N‐methyl‐D‐aspartate (NMDA, 5 μM, Tocris), to activate NMDARs, were in the presence (Figure 6a–c) or absence (Figures 6d–f and 7) of NBQX (10 μM, Sigma) to block AMPA/KARs. In some experiments prosaptide TX 14(a) (10 μM, a GPR37L1 agonist, AnaSpec), D‐serine (50 μM, an NMDAR co‐agonist, Tocris) or TNF‐α (10 ng mL^−1^, R & D) were used.

To record the glutamate uptake current (Figure 5), astrocytes were voltage‐clamped near their resting potential (∼–90 mV). Pharmacological blockers were present which increased the cell's membrane resistance and reduced currents that might be evoked by changes of [K^+^]_o_ occurring in response to a rise of extracellular glutamate concentration: TTX (150 nM, Tocris), the GABA_A_ receptor blocker bicuculline (10 μM, Sigma), NMDAR blockers (D‐AP5, 50 μM, Tocris; (+)MK‐801, 10 μM, Sigma; 5,7‐dichlorokynurenate, 10 μM, Sigma), the AMPAR/KAR blocker (disodium NBQX, 10 μM, Sigma) and the inwardly‐rectifying potassium channel blocker (BaCl_2_, 200 μM, Sigma). d‐aspartate (200 μM, Sigma) was added to evoke a glutamate transporter current in astrocytes (Gundersen, Shupliakov, Brodin, Ottersen, & Storm‐Mathisen, 1995). A non‐transported glial glutamate transporter blocker TFB‐TBOA (10 μM, Tocris) was used to block this.

### 2.8 Intracellular solutions

Neurons were patch‐clamped using 130 mM Cs‐gluconate, 4 mM NaCl, 10 mM HEPES, 0.1 mM CaCl_2_, 1 mM EGTA, 2 mM MgATP and 0.5 mM Na_2_GTP (pH 7.1–7.2 adjusted with CsOH, osmolarity ∼285 mOsm L^−1^). Alexa Fluor‐488 (40 μM) was added on the day of the experiment. For astrocytes, the electrode solution was 130 mM K‐gluconate, 4 mM NaCl, 10 mM HEPES, 1 mM CaCl_2_, 10 mM EGTA, 2 mM MgATP, 0.5 mM Na_2_GTP (pH 7.1–7.2 adjusted with KOH, osmolarity ∼285 mOsm L^−1^). Alexa Fluor‐488 or −594 (20 μM) was added on the day of the experiment.

### 2.9 Field excitatory postsynaptic current recordings

Thick‐walled glass electrodes, filled with HEPES‐based aCSF, were connected to a stimulator, and stimuli (in 20 V steps from 0–100 V) were applied with the electrode tip close to the CA3 pyramidal axon initial segments in hippocampal slices, to evoke field excitatory postsynaptic currents (fEPSCs, recorded in voltage‐clamp mode) that were recorded using an aCSF‐filled pipette near the apical dendrites of the CA1 pyramidal neurons.

### 2.10 Image analysis

Sections were examined in a LEICA SPE confocal microscope and micrographs were analyzed with ImageJ software (NIH), unless otherwise stated.

### 2.11 RNA purification and quantitative real‐time PCR

Total RNA was extracted from hippocampus with Trizol (Invitrogen), treated with RQ1 DNase (Promega) and complementary DNA was synthesized from 0.5 µg RNA with the High‐Capacity cDNA Reverse Transcription Kit (Applied Biosystems). Target cDNA levels were determined by RT‐PCR with the RealPlex unit (Eppendorf) using SYBR Green (Takyon No Rox SYBR MasterMix blue dTTP). Amplification assays were performed in 20 µL reaction mixtures containing Takyon No Rox SYBR MasterMix, 200 nM forward and reverse primers and cDNA. PCR was conducted over 35 cycles of 95°C for 15 s, 60°C for 60 s, preceded by an initial denaturation cycle at 95°C for 10 min. Actin cDNA levels were used to normalize the amount of cDNA. Quantification employed the comparative Δ‐ΔCt method (Pfaffl, 2001). Forward and reverse PCR primers were, respectively:

$$\left(\text{Actin}\right)\;5^{\prime }-\text{TCCACACCCGCCACCAG}\;\text{and}\;5^{\prime }-\text{TGACCCATTCCCACCATCACA};$$

$$\left(\text{Glast}\right)\;5^{\prime }-\text{CCAAACACAAAGGAGGGCTC}\;\text{and}\;5^{\prime }-\text{ACAGGATCGTTTGCCACCTA};$$

$$\left(\text{GLT}1\right)\;5^{\prime }-\text{TTGCTGGCATATTCCAAGCC}\;\text{and}\;5^{\prime }-\text{TTAATGGTTGCTCCGACTGG}.$$

### 2.12 Western Blots

Hippocampi were lysed in RIPA buffer with 1× complete protease inhibitors (cOmplete, EDTA‐free Protease Inhibitor Cocktail, Roche). Protein levels were assessed with a Bradford assay with bovine serum albumin as the standard.

About 20 µg of denatured proteins in Laemmli buffer were separated by 8% SDS‐polyacrylamide gel electrophoresis and blotted onto PVDF membranes (GE Healthcare). Nonspecific binding was blocked with PBS‐0.1% Tween‐20 (PBST) with 3% (w/v) non‐fat dried milk (Sigma), for 1 hr at 20–25°C. Membranes were incubated overnight at 4°C in PBST with 3% milk and primary antibodies: anti‐GLT1 (1/500, Millipore AB1783), mouse anti‐β‐Actin (1:5000, Sigma A1978). Membranes were then incubated at 20–25°C for 1 hr in PBST/3% milk with POD‐conjugated recombinant protein‐A (Invitrogen). Protein bands were detected by enhanced chemiluminescence (GE Healthcare) and quantified by densitometry with ImageJ. Protein levels were normalized to those of β‐actin controls.

### 2.13 Chemical Ischemia

Hippocampal slices (270 μm) from P14‐P16 *Gpr37l1* ^+/+^ or *Gpr37l1* ^–/–^ littermates were allowed to recover for 40 min before being incubated for 30 min at 37°C in (1) control solution containing 124 mM NaCl, 26 mM NaHCO_3_, 10 mM glucose, 2.5 mM KCl, 2 mM CaCl_2_, 1 mM NaH_2_PO_4_, 1 mM MgCl_2_, pH 7.2–7.4, gassed with 95% O_2_/5% CO_2_, or (2) ischemic solution with the glucose replaced by 7 mM sucrose, gassed with 95% N_2_/5% CO_2_, and with 2 mM iodoacetate and 25 μM antimycin added to block glycolysis and oxidative phosphorylation, respectively. Propidium iodide (PI, 7.5 μM) was added to label dead cells by binding to DNA/RNA. Slices were then fixed for 1 hr in 4% PFA and immunohistochemistry for NEUN and GFAP was performed. Two slices per condition were analyzed. Four z‐stacks were generated per slice and PI‐labelled cells were counted in the stratum radiatum and pyramidal cell layer, with experimenters blind to the mouse genotype. Images were 275 µm square and the z‐stack depth was 25 µm (z‐step = 0.5 µm). Gain and offset settings were identical for all slices in each experiment.

### 2.14 Middle cerebral artery occlusion

Brains from mice that had experienced middle cerebral artery occlusion (MCAO) were kindly donated by Kaylene Young. The ISH signal for *Gpr37l1* was quantified using ImageJ (Figure 8).

### 2.15 Mouse behaviour

Mice were handled daily for 1 week to habituate them prior to behavioural tests. They were left in their home cages in the behaviour room for 30 min before initiating tests. Trials were recorded using a top‐view video camera and white noise (50 dB) was played during the tests.

#### 2.15.1 Open‐field test

Mice were allowed to explore a 30 cm^2^ arena for 30 min and tracked with ActualTrack software. Total distance travelled and time spent in the centre was calculated.

#### 2.15.2 Novel‐object recognition

Mice were placed in the arena for 5 min before being familiarized for 10 min with two identical objects. After a 10‐min delay, mice were tested for 10 min by placing them in the arena with one of the original objects replaced by a novel object (NOR). 24 hr later mice were tested again for 10 min with one familiar and one new object (NOR + 24). The times spent inspecting the novel and familiar objects were assessed with the ActualTrack software. The discrimination index (DI) was calculated as: DI = {(time spent with novel object) minus (time spent with familiar object)}/(total time spent with both objects).

### 2.16 Rotarod

About 2–3 months or 6‐month‐old mice were familiarized with the rotarod for three trials at a constant speed of 4 rpm. They were then tested for 3 days, with three trials/day, at an accelerating speed from 4 to 40 rpm for up to 5 min. The latency to fall was recorded.

### 2.17 Statistics

Statistical significance was determined with GraphPad Prism (GraphPad Software, CA, USA) and OriginPro software. Data normality was assessed using Kolmogorov–Smirnov tests. Data are presented as mean ± SEM. Data were corrected for multiple comparisons using a procedure equivalent to Holm‐Bonferroni (for N comparisons, the most significant *p* value is multiplied by N, the 2nd most significant by N‐1, the 3rd most significant by N‐2, etc.; corrected *p* values are regarded as significant if they are <0.05).

## 3 RESULTS

### 3.1 *Gpr37l1* is expressed in astrocytes and some oligodendrocyte precursors

**Figure 1:**
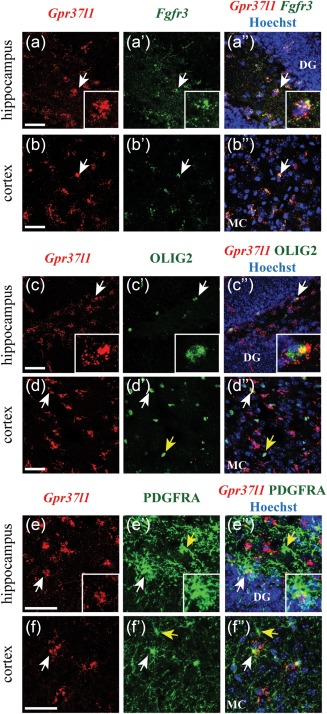
*Gpr37l1* is expressed in astrocytes and OPs. Cells expressing *Gpr37l1* transcripts were distributed throughout all regions of the adult forebrain including hippocampus (a, c, e), and cortex (b, d, f). Confocal fluorescent double ISH showed expression of *Gpr37l1* in *Fgfr3*‐positive astrocytes in hippocampus (a) and cortex (b). ISH for *Gpr37l1* followed by immunohistochemistry demonstrated that *Gpr37l1* expression co‐localized with OLIG2 (c, d) and PDGFRA (e, f). White arrows: double‐positive cells; yellow arrows: single OLIG2‐ or PDGFRA‐positive cells. DG: dentate gyrus, MC: motor cortex. Scale: 50 µm

We combined in situ hybridization (ISH) with immunolabelling to determine which cells express *Gpr37l1* in different brain areas. *Gpr37l1* is widely expressed in the hippocampus (Figure 1a,c,e), cerebral cortex (Figure 1b,d,f) and corpus callosum (Supporting Information Figure 1). From our ISH and the Allen Brain Atlas, expression of *Gpr37l1* in the hippocampus is similar in CA1, CA3 and dentate gyrus (http://mouse.brain‐map.org/gene/show/82624). Double ISH detected *Gpr37l1* mRNA in *Fgfr3*‐positive astrocytes in both grey and white matter (Figure 1a,b and Supporting Information Figure 1a). *Gpr37l1* is also expressed throughout the brain in OL‐lineage cells immunolabelled for OLIG2 (Figure 1c,d, Supporting Information Figure 1b) and in OPs immunolabelled for PDGFRA (Figure 1e,f, Supporting Information Figure 1c). Approximately 95, 91, and 82% of *Fgfr3^+^* astrocytes were *Gpr37l1^+^* in the cortex, hippocampus and corpus callosum, respectively, while ∼23, ∼30, and ∼25% of PDGFRA^+^ cells in these regions coexpressed *Gpr37l1*. In the cortex, hippocampus and corpus callosum the proportions of *Gpr37l1^+^* cells that co‐expressed *Fgfr3* were ∼68, ∼69, and ∼68%, respectively, while the proportions of *Gpr37l1* ^+^ cells that co‐expressed PDGFRA were ∼32, ∼32, and ∼31%. Thus, all *Gpr37l1‐*expressing cells appear to be either *Fgfr3^+^* astrocytes or PDGFRA^+^ OPs. Most or all (>90%) grey matter astrocytes and a substantial fraction of white matter astrocytes (>80%) express *Gpr37l1*, as well as a minority (∼25%) of OPs in grey and white matter.

To confirm these results, we used *Gpr37l1*‐*LacZ* heterozygous mice in which a *LacZ* cassette was inserted into the first exon of the *Gpr37l1* gene (inactivating the protein product). Immunolabelling for β‐galactosidase confirmed that *Gpr37l1‐LacZ* was expressed in PDGFRA‐positive OPs in the cortex (Supporting Information Figure 2a) but not in CC1^+^ mature OLs, NEUN^+^ neurons or IBA1^+^ microglia (Supporting Information Figure 2b–d). In addition, *Gpr37l1‐LacZ* was expressed in the cerebellum in Bergman glia and in OL‐lineage cells identified by SOX10 immunolabelling (Supporting Information Figure 2e,f).

**Figure 2:**
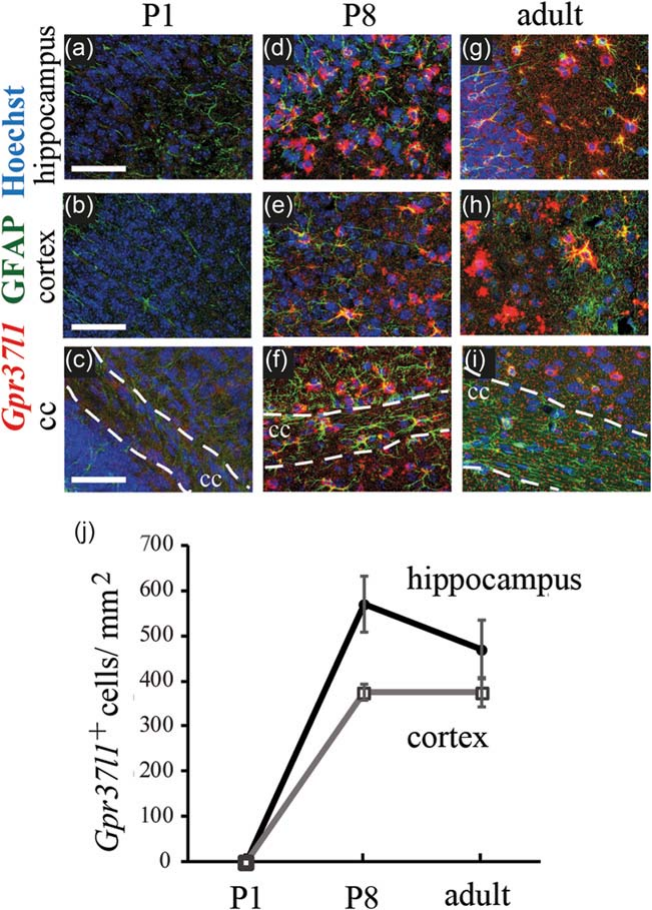
Expression of *Gpr37l1* is developmentally‐regulated. Expression of *Gpr37l1* at different postnatal stages (P1, P8, adult) using ISH followed by immunolabelling. At P1, *Gpr37l1* was not expressed in the brain (a–c). *Gpr37l1* expression in GFAP‐labelled astrocytes in the brain started at ∼P8 in the hippocampus, cortex and corpus callosum (d–f). In the adult (g–i), *Gpr37l1* expression in astrocytes was maintained. (j) Number of cells expressing *Gpr37l1* in the cortex and the hippocampus of P1, P8 and adult mice. Scale bars in (a‐i): 50 µm

Expression of *Gpr37l1* was developmentally regulated. At postnatal day 1 (P1), *Gpr37l1* mRNA was not detectable in any brain area examined (Figure 2a–c) but at P8 *Gpr37l1* was strongly expressed in both astrocytes (Figure 2d–f) and OPs (not shown). At P15 (not shown) and during adulthood, *Gpr37l1* expression in astrocytes (Figure 2g–i) and OPs (not shown) remained at high levels. Thus, GPR37L1 might have a functional role from the period of synaptogenesis and the onset of myelination through to adulthood (Figure 2j).

### 3.2 *Gpr37l1* and *Gpr37* are expressed in different cells

**Figure 3:**
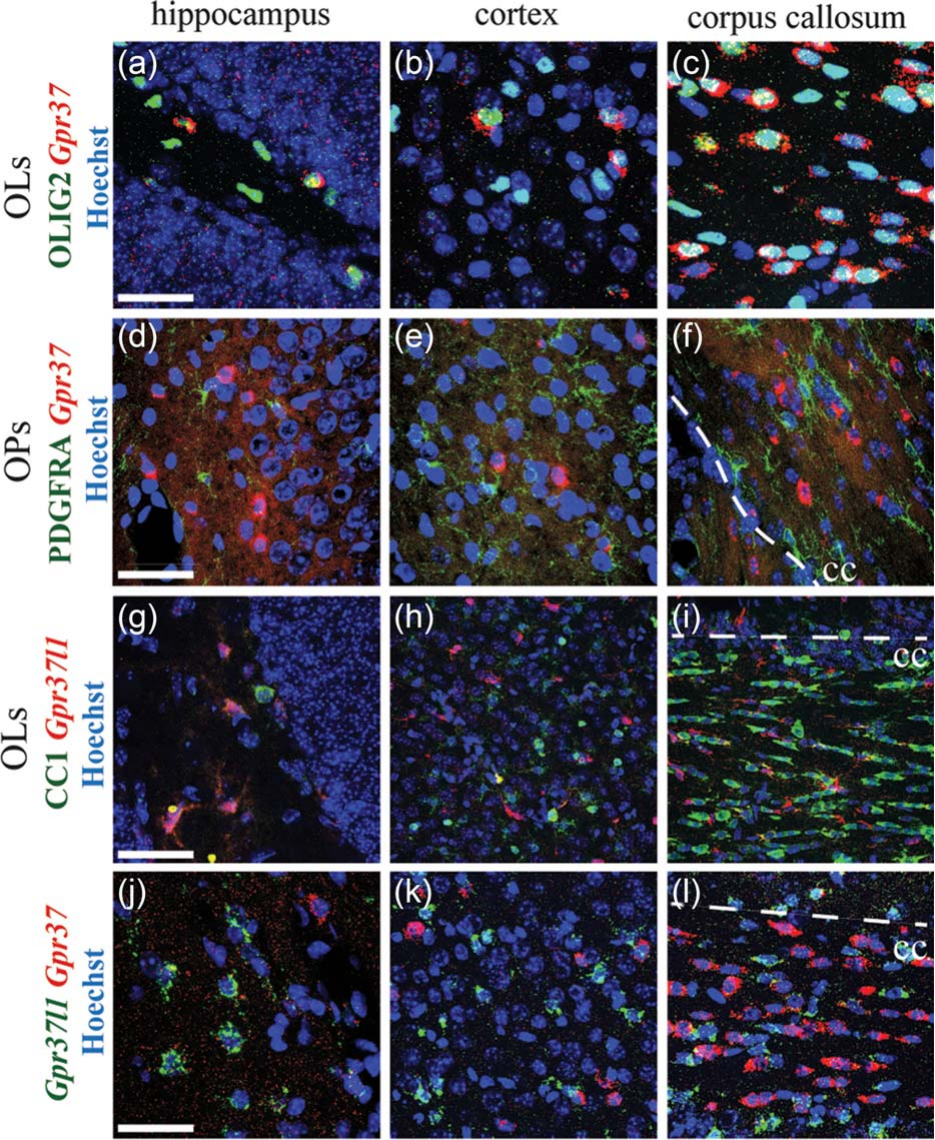
*Gpr37l1* and *Gpr37* are expressed in mutually exclusive cell populations. Cells expressing *Gpr37* transcripts were mostly found in subcortical areas (hypothalamus and thalamus) and in corpus callosum; fewer *Gpr37^+^* cells were present in cortex and hippocampus. Fluorescent ISH revealed expression of *Gpr37* in OLIG2^+^ OL lineage cells (a–c), but not in OPs expressing PDGFRA (d–f). Conversely, immunolabelling of *Gpr37l1*‐*LacZ* heterozygous mice for β‐galactosidase showed that *Gpr37l1* is not expressed in CC1‐positive mature OLs (g–i). Fluorescent double‐ISH demonstrates that *Gpr37l1* and *Gpr37* are expressed in different cells in hippocampus (j), cortex (k) and corpus callosum (L). Dotted lines: boundary between cortical grey matter and corpus callosum (cc). Scale: 50 µm

GPR37L1 and its close relative GPR37 share 48% amino acid identity in human (Valdenaire et al., 1998). ISH for *Gpr37* mRNA showed that *Gpr37* was expressed in many cells in subcortical structures such as the hypothalamus and thalamus as well as in the corpus callosum, and in smaller numbers of cells in the cortex and hippocampus (Figure 3). *Gpr37* was mostly in OLIG2^+^ oligodendrocyte (OL)‐lineage cells (Figure 3a–c) but not in PDGFRA^+^ cells (Figure 3d–f), suggesting that mature OLs but not OPs express *Gpr37*. We observed no expression of *Gpr37* in GFAP^+^ astrocytes (not shown). Occasionally, *Gpr37* expression was seen in some NEUN^+^ neurons but not in IBA1^+^ microglia (not shown).

In contrast to *Gpr37*, *Gpr37l1* is not expressed in CC1^+^ mature OLs, judging by immunolabelling of *Gpr37l1‐LacZ* heterozygous mice for β‐galactosidase (Figure 3g–i; Supporting Information Figure 2b). Thus, *Gpr37l1* and *Gpr37* are expressed in complementary cell types, *Gpr37l1* being highly expressed in astrocytes and OPs whereas *Gpr37* is expressed in mature OLs and some neurons. Similarly, double ISH for both *Gpr37l1* and *Gpr37* revealed that *Gpr37l1* and *Gpr37* were expressed in non‐overlapping cell populations in the hippocampus (Figure 3j), cortex (Figure 3k) and corpus callosum (Figure 3l). In the following, we focus on the functional significance of *Gpr37l1* expression in astrocytes.

### 3.3 *Gpr37l1* KO has little effect on cell or synapse number, or motor function

We assessed the phenotypic consequences of *Gpr37l1* knockout (KO). Deletion of *Gpr37l1* did not trigger gliosis (assessed by screening for increased expression of GFAP in astrocytes or IBA1 in microglia) in 1‐month‐old mice (Supporting Information Figure 3a). Although *Gpr37l1* is expressed in OPs, its deletion did not affect *Mbp* expression (Supporting Information Figure 3b) or the number of PDGFRA^+^ OPs in the corpus callosum (Supporting Information Figure 3c, *p* = 0.38). Furthermore, *Gpr37* expression was not changed in *Gpr37l1* ^–/–^ mice (Supporting Information Figure 7), implying no compensation for the loss of *Gpr37l1* by upregulation of *Gpr37*.

Previous reports claimed that knocking‐out *Gpr37l1* resulted in precocious cerebellar development and enhanced motor skills (Marazziti et al., 2013). However, surprisingly, we found that locomotor activity and exploratory behaviour of our *Gpr37l1* knockouts were similar to wild‐type mice in the open‐field test (3‐month old mice, *t* test for total distance travelled and time spent in the centre, *p* = 0.96 and 0.47, respectively), novel object recognition test (3‐month‐old mice, two‐way ANOVA, *p* = 0.29) and rotarod (3‐ and 6‐month‐old mice; two‐way ANOVA, *p* = 0.77; Supporting Information Figure 3d–f). We therefore searched for more subtle functions of *Gpr37l1* in physiology or pathology.

Astrocytes regulate neuronal and synaptic development, and neuronal activity (Allen, 2014). As expression of *Gpr37l1* during development (Figure 2) correlates with the time of synaptogenesis (Crain, Cotman, Taylor, & Lynch, 1973), we assessed whether *Gpr37l1* knockout affected hippocampal synapse formation. We found no difference between *Gpr37l1* ^–/–^ and *Gpr37l1* ^+/+^ mice in the number of excitatory synapses identified with PSD95 and vGLUT2 antibodies (PSD95/vGLUT2 *t* test, *p* = 0.37, Supporting Information Figure 4a–d), or in the number of inhibitory synapses identified with vGAT antibodies (*t* test, *p* = 0.48, Supporting Information Figure 4e,f). There was also no difference in astrocyte morphology (from GFAP staining), the number of GFAP‐expressing astrocytes (*p* = 0.65), the area of each astrocyte in maximum intensity projections (*p* = 0.69), or the mean GFAP intensity (*p* = 0.29) (Supporting Information Figure 5a–e).

### 3.4 *Gpr37l1* KO does not alter the input resistance of astrocytes or neurons or neuronal excitability

GPR37L1 can protect astrocytes against oxidative stress (Meyer et al., 2013), and we show below that it also protects neurons in ischemia. This suggests that the membrane properties or response to glutamate of neurons and astrocytes might be modulated by GPR37L1.

**Figure 4:**
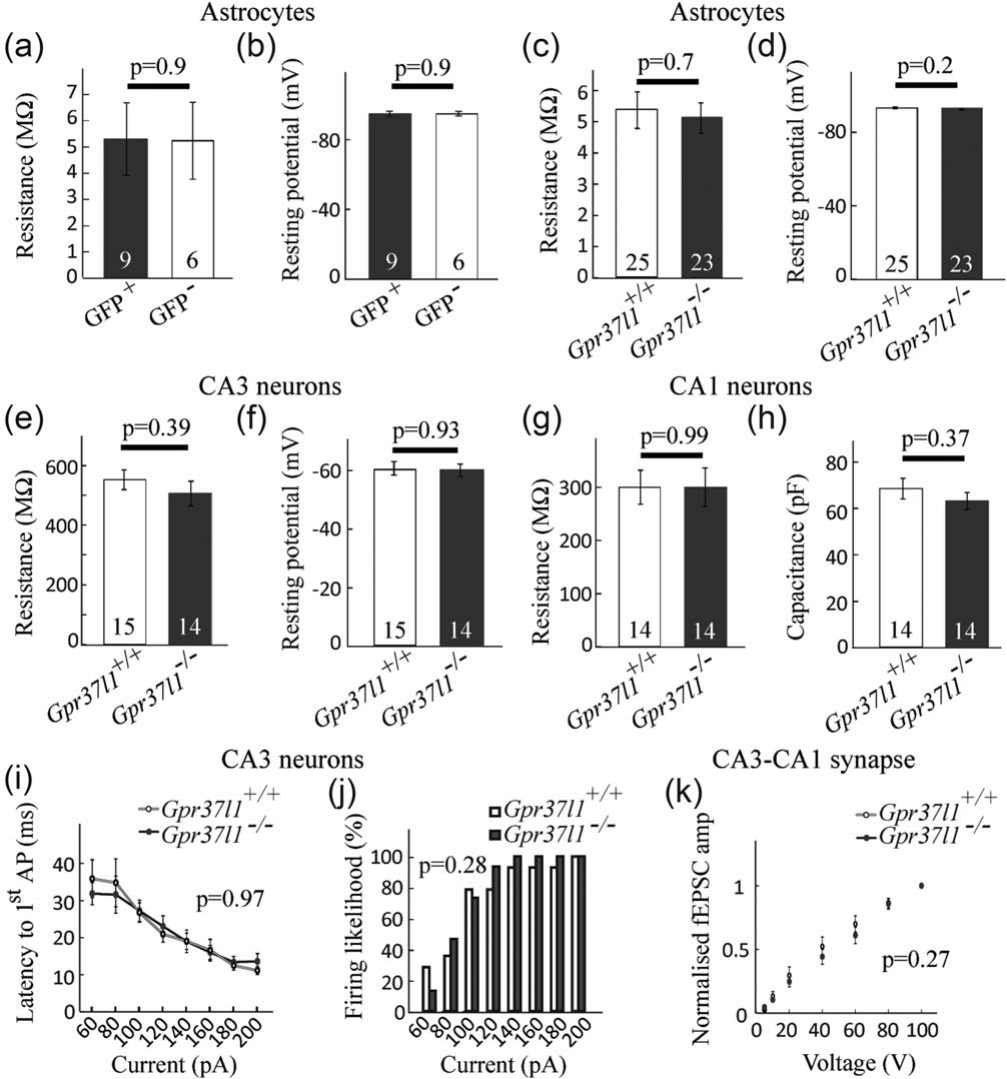
Resting electrical properties of astrocytes and neurons are not affected by *Gpr37l1* expression. (a, b) Astrocytes expressing or lacking GFP in hippocampal slices from the *Gfp37l1‐GFP* mouse have similar (a) membrane resistance, and (b) resting potential (number of cells on bars). Astrocytes in hippocampal slices from wild type and *Gpr37l1* knock‐out mice have similar (c) membrane resistance and (d) resting potential. (e, f) CA3 pyramidal cells in hippocampal slices from wild type and *Gpr37l1* knock‐out mice have similar (e) membrane resistance and (f) resting potential. (g, h) CA1 pyramidal cells in hippocampal slices from wild type and *Gpr37l1* knock‐out mice have similar (g) membrane resistance and (h) capacitance (used to normalise drug‐evoked currents in Figure 5; resting potential was not studied as the internal solution contained Cs^+^). (i, j) Excitability of CA3 neurons in slices from wild type and *Gpr37l1* knock‐out mice. (i) Latency to first action potential as a function of current injected into CA3 pyramidal neurons (*Gpr37l1* ^+/+^ *n* = 15, *Gpr37l1* ^–/–^ *n* = 15). (j) Percentage of responses in (i) that showed action potentials as a function of injected current. (k) Field EPSCs evoked in area CA1 by applying stimuli to the Schaffer collaterals of CA3 axons, in 20 V steps from 0 to 100 V. Amplitudes of field EPSCs were normalized to the maximal response (at 100 V) for each slice (*Gpr37l1* ^+/+^ *n* = 8, *Gpr37l1* ^–/–^ *n* = 9)

Hippocampal astrocytes expressing or lacking *Gpr37l1* expression, as defined by fluorescent detection of GFP in *Gpr37l1‐GFP* mice, did not differ in input resistance (Figure 4a, *p* = 0.9) or resting potential (Figure 4b, *p* = 0.9). Similarly, hippocampal astrocytes in *Gpr37l1* ^+/+^ and *Gpr37l1* ^–/–^ mice exhibited no difference in input resistance (Figure 4c, *p* = 0.7) or resting potential (Figure 4b, *p* = 0.2). CA3 and CA1 pyramidal neurons in *Gpr37l1* ^+/+^ and *Gpr37l1* ^–/–^ slices also had similar membrane resistance (Figure 4e,g, *p* = 0.39 and *p* = 0.99, respectively) and the resting potential of CA3 neurons was also unaffected by *Gpr37l1* knock‐out (Figure 4f, *p* = 0.93) (resting potentials of CA1 cells were not measured as they were patch‐clamped with a Cs^+^‐containing internal solution), as was the capacitance of CA1 neurons (Figure 4h, *p* = 0.37).

We assessed the excitability of CA3 pyramidal neurons using whole‐cell current‐clamp recordings in slices from *Gpr37l1* ^–/–^ and *Gpr37l^+/+^* mice. We found no difference between the *Gpr37l1* ^–/–^ and *Gpr37l^+/+^* cells for the latency to the first action potential evoked by depolarizing current injected at the soma (one‐way ANOVA, *p* = 0.97, Figure 4i), nor was there a difference in the firing probability as a function of injected current (Kolmogorov–Smirnov test *p* = 0.28, Figure 4j). Furthermore, when recording stimulation‐evoked field excitatory postsynaptic currents (fEPSCs) generated in CA1 in response to Schaffer collateral stimulation, the dependence of fEPSC amplitude on stimulus magnitude was similar in *Gpr37l1* ^–/–^ and *Gpr37l1* ^+/+^ slices (Figure 4k, Kolmogorov–Smirnov test *p* = 0.27, *n* = 8 for *Gpr37l1* ^+/+^ and *n* = 9 for *Gpr37l1* ^–/–^). Thus, there is no difference in the excitability of CA3 neurons or their axons in *Gpr37l1* ^+/+^ and *Gpr37l1* ^–/–^ hippocampal slices.

### 3.5 Prosaptide‐evoked GPR37L1 signalling inhibits astrocyte glutamate uptake

Any alteration by GPR37L1 of the clearance of glutamate by glial glutamate transporters could change tonic excitation and synaptic currents mediated by glutamate receptors. A change of glutamate transport rate might also alter the extracellular glutamate concentration reached in ischemia when transporters reverse and release glutamate (Rossi, Oshima, & Attwell, 2000). Such a change of glutamate release should alter NMDA receptor (NMDAR)‐mediated cell death in ischemia (Brassai, Suvanjeiev, Ban, & Lakatos, 2015; Vornov and Coyle, 1991) and thus contribute to the neuroprotective effect in ischemia of GPR37L1 and prosaptide [see below and Morita et al. (2001)].

**Figure 5:**
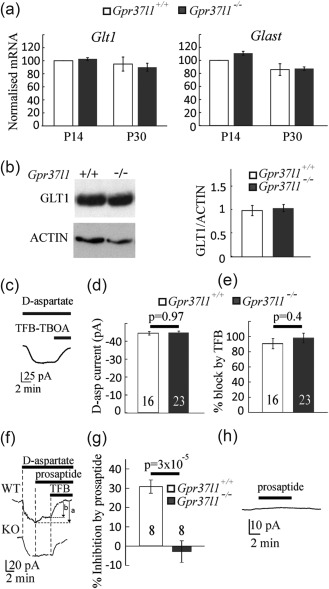
Assessment of glutamate uptake in astrocytes. (a) Expression of mRNA for the glutamate transporters *Glast* and *Glt‐1* assessed by RT‐PCR in hippocampus from P14 and P30 *Gpr37l1* ^+/+^ and *Gpr37l1* ^–/–^ mice. Data are mean ± s.e.m from four experiments. (B) Expression of GLT‐1 analyzed by Western blot in hippocampus from P14 *Gpr37l1* ^+/+^ and *Gpr37l1* ^–/–^ mice (quantified relative to actin). Data are mean ± s.e.m of four experiments. (c–e) Glutamate uptake current in astrocytes (number of cells on bars). (c) Example of a d‐aspartate (200 µM)‐evoked current in an astrocyte at −100 mV, and its inhibition by TFB‐TBOA (10 µM). (d) Current magnitude. (e) Percentage inhibition of the current by TFB‐TBOA. (f–h) Activation of GPR37L1 inhibits glutamate transport in astrocytes. (f) The d‐aspartate (200 µM)‐evoked inward current is partly inhibited by prosaptide (10 µM, see the difference between the arrows marked a and b in the *Gpr37l1* ^+/+^ cell but not in the *Gpr37l1* ^–/–^ cell). (g) Quantification of the inhibition of the d‐aspartate evoked current by prosaptide. (h) Prosaptide does not evoke a current in the absence of d‐aspartate (in the WT)

To test this hypothesis, we first compared the expression levels of the glutamate transporters, GLT‐1 and GLAST, expressed in astrocytes, using hippocampal extracts from *Gpr37l1* ^+/+^ and *Gpr37l1* ^–/–^ mice. Quantitative PCR showed that *Glast* and *Glt1* mRNA levels were similar in *Gpr37l1* ^+/+^ and *Gpr37l1* ^–/–^ mice at P14 and P40 (Figure 5a, two‐way ANOVA, *Glt1 p* = 0.56, *Glast p* = 0.36). Similarly, GLT‐1 protein expression was similar in *Gpr37l1* ^+/+^ and *Gpr37l1* ^–/–^ mice (Figure 5b, *t* test *p* = 0.7). We then compared the magnitude of the glutamate transporter current in *Gpr37l1* ^+/+^ and *Gpr37l1* ^–/–^ hippocampal astrocytes. Astrocytes in the stratum radiatum were whole‐cell voltage‐clamped (near their resting potential) and responses to d‐aspartate (200 μM), a substrate for glutamate transporters (Gundersen et al., 1995), were recorded in the presence and absence of the glutamate transporter blocker TFB‐TBOA (10 μM, Figure 5c). Blockers of NMDARs, AMPARs, GABA_A_Rs, voltage‐gated Na^+^ channels and inwardly rectifying potassium channels were also present throughout the experiment (see Materials and Methods).

In the absence of prosaptide, the glutamate uptake current was similar in *Gpr37l1* ^–/–^ and *Gpr37l1* ^+/+^ astrocytes (Figure 5d, *p* = 0.97). TFB‐TBOA (10 μM), which blocks both GLT‐1 and GLAST transporters (Shimamoto et al., 2004), blocked the d‐aspartate evoked current in both *Gpr37l1* ^+/+^ and *Gpr37l1* ^–/–^ astrocytes (Figure 5e, *p* = 0.4), confirming that the current is generated by glutamate transporters. The lack of a difference in glutamate transporter current with GPR37L1 knocked out could reflect GPR37L1 not being activated under physiological conditions, since it is known that the expression and release of prosaposin are up‐regulated following ischemia (Costain et al., 2010; Hiraiwa et al., 2003). We therefore investigated the effect of prosaptide on the glutamate transporter current evoked by d‐aspartate (200 μM), to test whether it modulates the uptake current in the presence or absence of GPR37L1.

Adding prosaptide (10 μM), at the peak of the d‐aspartate‐evoked current, significantly reduced the uptake current in *Gpr37l1* ^+/+^ astrocytes but not in *Gpr37l1* ^–/–^ astrocytes (Figure 5f,g, inhibition 31% ± 3% for *Gpr37l1* ^+/+^ and −2% ± 6% for *Gpr37l1* ^–/–^, significantly different, *p* = 3 × 10^−5^). Importantly, prosaptide alone (without D‐aspartate) failed to generate any current in either *Gpr37l1* ^+/+^ or *Gpr37l1* ^–/–^ astrocytes (Figure 5h), showing that the outward prosaptide‐evoked current in the presence of D‐aspartate reflects suppression of the inward uptake current and not an effect on the baseline membrane current. The mean current generated by prosaptide alone was −1 ± 2 pA for three *Gpr37l1* ^+/+^ astrocytes and 0.2 ± 1.0 for four *Gpr37l1^–^* ^/–^ astrocytes (not significantly different from zero, *p* = 0.5 and 0.9, respectively). The inhibition of glutamate transporters by prosaptide in *Gpr37l1* ^+/+^ astrocytes is presumably mediated by GPR37L1 receptors in the astrocytes themselves and cannot reflect prosaptide acting on the related GPR37 receptor because it had no effect in *Gpr37l1* ^–/–^ slices.

### 3.6 GPR37L1 signalling decreases neuronal responses to prolonged NMDA application

**Figure 6:**
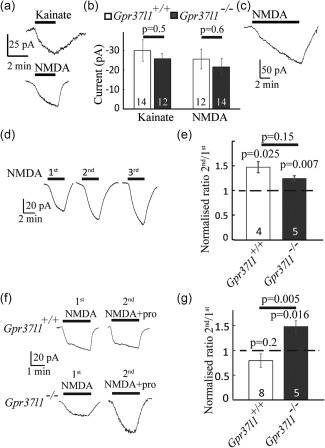
Suppression of potentiation of repeated NMDAR responses in CA1 pyramidal cells by GPR37L1. (a, b) Current responses to brief application of (a) kainate (3 µM, to activate kainate and AMPA receptors) and (b) NMDA (5 µM) at −30 mV were similar in *Gpr37l1* ^+/+^ and *Gpr37l1* ^–/–^ slices. (c) Prolonged NMDA application evokes a slowly increasing current. (d) Repeated (at 4‐min intervals) application of NMDA (5 μM) also evoked a gradual increase in response magnitude. (e) Quantification of the increase in (d) in *Gpr37l1* ^+/+^ and *Gpr37l1* ^–/–^ cells. In both *Gpr37l1* ^+/+^ and *Gpr37l1* ^–/–^ slices, the response to the second application of NMDA was larger than the first. *p* values above bars in (e) and (g) compare with a ratio of 1. (f) As in (d) but with prosaptide (10 μM) present for the second application. (g) Prosaptide inhibited potentiation of the NMDA current in *Gpr37l1* ^+/+^ (ratio not significantly different from 1) without affecting the potentiation in the *Gpr37l1* ^–/–^ [ratio ∼1.5, and not significantly different from that in (e), *p* = 0.15]. Numbers of cells are on bars. All recordings were in the presence of TTX (150 nM) and picrotoxin (100 µM); in (a) kainate was applied in the presence of D‐AP5 (5 μM), while in (a) and (c) NMDA was applied with NBQX (10 μM) also present

Although *Gpr37l1* knock‐out did not affect the intrinsic excitability of neurons (Figure 4), it could in principle affect synaptic transmission between neurons. Although *Gpr37l1* is expressed only in glia, gliotransmitters released from astrocytes, such as D‐serine and TNFα, have been shown to modulate glutamate‐gated currents in neurons (Henneberger, Papouin, Oliet, & Rusakov, 2010; Shigetomi, Jackson‐Weaver, Huckstepp, O'Dell, & Khakh, 2013; Stellwagen and Malenka, 2006). We recorded responses to kainate (3 µM, to activate AMPA/KA receptors, in the presence of the NMDAR blocker D‐AP5) or to NMDA (5 µM, in the presence of the AMPA/KA receptor blocker NBQX) in CA1 pyramidal neurons voltage‐clamped at −30 mV (to promote Mg^2+^ unbinding from NMDAR channels). No difference was seen between the responses of neurons in *Gpr37l1^–/–^* or *Gpr37l1* ^+/+^ slices to a single brief application of KA or NMDA (Figure 6a,b, *p* = 0.5 for KA and *p* = 0.6 for NMDA).

Prolonged application of NMDA to *Gpr37l1^–/–^* or *Gpr37l1* ^+/+^ slices evoked a slowly increasing inward current in CA1 pyramidal neurons, suggesting a sensitization of the NMDA response with time (Figure 6c). Indeed, repeated brief applications of 5 µM NMDA (3 times for 3 minutes at 4 minute intervals) resulted in a progressive increase in the current evoked (Figure 6d). In neurons from both *Gpr37l1* ^+/+^ and *Gpr37l1* ^–/–^ slices, the response to the second application of NMDA was larger than the first (mean ratio 1.5 ± 0.1, one sample *t* test, significantly >1, *p* = 0.025 for *Gpr37l1* ^+/+^ cells; mean ratio 1.3 ± 0.1, *p* = 0.007, for *Gpr37l1* ^–/–^ cells) (Figure 6e). This potentiation was not significantly different in *Gpr37l1* ^+/+^ and *Gpr37l1* ^–/–^ slices (unpaired *t* test *p* = 0.15).

When prosaptide (10 μM) was bath‐applied prior to and during the second application of 5 μM NMDA, the potentiation of the NMDA current was blocked in neurons in *Gpr37l1* ^+/+^ slices (Figure 6f,g; mean ratio 0.79 ± 0.13, not significantly different from 1, *p* = 0.2), without affecting the potentiation of the NMDA response in neurons in *Gpr37l1* ^–/–^ slices (mean ratio 1.48 ± 0.12, significantly different from 1, *p* = 0.016, and not significantly different in the presence or absence of prosaptide, *p* = 0.15). The potentiation in *Gpr37l1* ^–/–^ slices was significantly greater than in *Gpr37l1* ^+/+^ slices, *p* = 0.005). Thus, GPR37L1‐mediated signalling in astrocytes decreases the neuronal NMDAR response during prolonged activation of NMDARs. This could provide a neuroprotective mechanism when both glutamate and prosaposin are released during ischemia.

### 3.7 How does astrocyte GPR37L1 regulate neuronal NMDAR responses?

Because GPR37L1 is present in astrocytes (and OPs, although these receptors may be less well positioned to regulate NMDAR responses), while the NMDAR responses recorded are from neurons, a signal must pass from astrocytes to neurons to alter the NMDAR response when GPR37L1 is activated by prosaptide. We tested whether the gliotransmitters d‐serine or TNF‐α mediate this effect.

**Figure 7:**
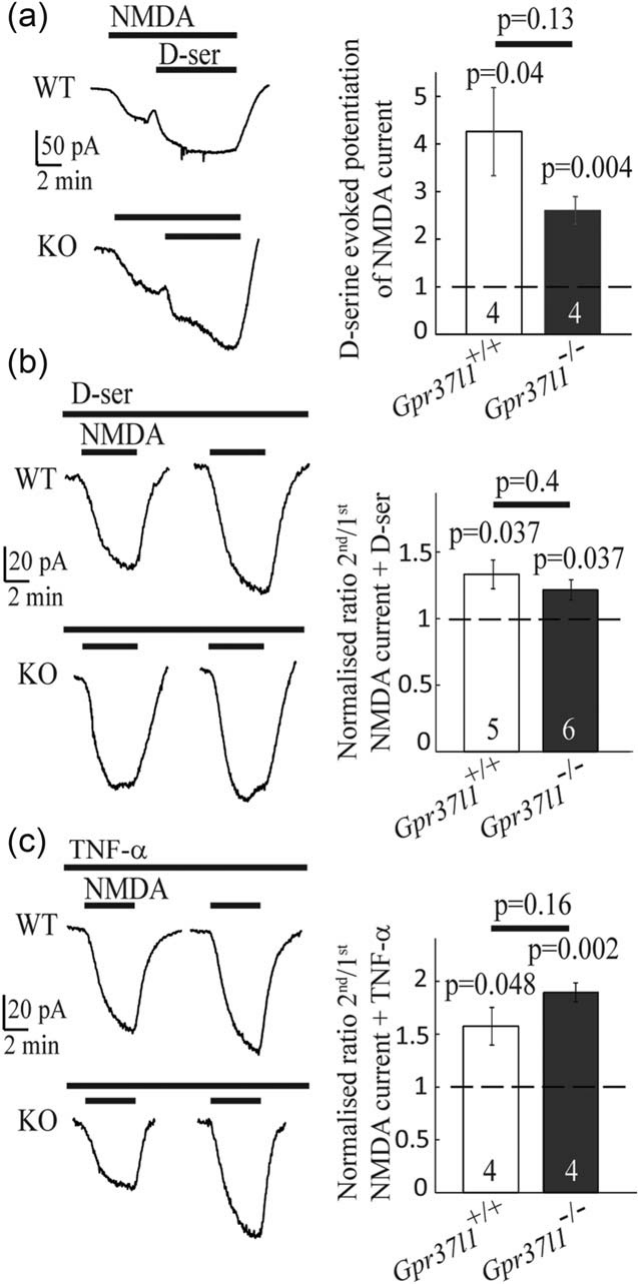
The gliotransmitters d‐serine and TNF‐α do not mediate the potentiation of repeated NMDAR responses by GPR37L1. (a) d‐serine potentiated the NMDA (5 µM)‐evoked response (numbers of cells on bars; *p* values above bars compare with a ratio of 1). (b) Bath application of 50 μM d‐serine did not prevent potentiation of the current in response to the second application of NMDA. (c) As for (b), but with TNF‐α (10 ng mL^−1^) present throughout. TNF‐α did not prevent potentiation of the current in response to the second NMDA application

Activation of NMDARs requires the binding of glutamate and a co‐agonist, either glycine or d‐serine (Johnson and Ascher, 1987; Papouin et al., 2012; Zhang et al., 2014). Increasing the concentration of glycine or D‐serine potentiates the NMDAR‐evoked response (Henneberger et al., 2010; Johnson and Ascher, 1987; Kang et al., 2013; Rosenberg et al., 2013), because the glycine/D‐serine binding site is not saturated in cortical brain slices (Fossat et al., 2012), indicating that changes of the NMDA response can occur if release of d‐serine (or glycine) is altered. Moreover, astrocyte‐derived d‐serine (Henneberger et al., 2010; Kang et al., 2013; Shigetomi et al., 2013) has been shown to co‐activate postsynaptic neuronal NMDARs. We therefore considered the possibility that the GPR37L1‐mediated inhibition of repeated NMDA responses (Figure 6e,f) might reflect a reduction in d‐serine release from astrocytes. Before testing this idea, we first confirmed that d‐serine could potentiate the NMDA response of CA1 neurons. Indeed, bath application of d‐serine (50 μM, in the presence of 5 μM NMDA), when applied at the peak of the current evoked by NMDA, further enhanced the current in CA1 pyramidal neurons of both *Gpr37l1* ^+/+^ and *Gpr37l1^–/–^* slices (current increased 4.3 ± 0.9 fold, *p* = 0.04 in *Gpr37l1* ^+/+^ and 2.6 ± 0.2 fold, *p* = 0.004 in *Gpr37l1^–/–^*; Figure 7a). This potentiation was not significantly different in the two genotypes (*p* = 0.13).

If increased release of d‐serine from astrocytes underlies the potentiation of the NMDA‐evoked response, bath perfusion of a saturating concentration of d‐serine throughout the experiment should prevent this effect. However, the presence of d‐serine (50 μM) throughout the experiment (Figure 7b) did not affect the potentiation of the second response to NMDA relative to the first response for neurons in either *Gpr37l1* ^+/+^ or *Gpr37l1^–/–^* slices (mean ratio 1.3 ± 0.1 for *Gpr37l1* ^+/+^, which is significantly different from 1, *p* = 0.037, and mean ratio 1.2 ± 0.1 for *Gpr37l1^–/–^*, which is significantly different from 1, one sample *t* test *p* = 0.037). This potentiation ratio was not different in the two genotypes (unpaired *t* test *p* = 0.4).

Alternatively, the GPR37L1‐mediated inhibition of NMDA‐evoked responses might involve a change in the release of tumour necrosis factor alpha (TNF‐α) from astrocytes, since TNF‐α also decreases (Glazner and Mattson 2000) or increases (Jara, Singh, Floden, & Combs, 2007; Marchetti, Klein, Schlett, Pfizenmaier, & Eisel, 2004) neuronal NMDA responses and modulates NMDAR‐mediated excitotoxicity (Jara et al., 2007; Marchetti et al., 2004). Astrocytes are a source of TNF‐α (Stellwagen and Malenka 2006). However, bath perfusion of TNF‐α (10 ng/ml) throughout the experiment (Figure 7c) did not alter the potentiation of the response to NMDA for neurons in either *Gpr37l1* ^+/+^ or *Gpr37l1^–/–^* slices (mean ratio = 1.6 ± 0.2 for *Gpr37l1* ^+/+^, significantly different from 1, *p* = 0.048, and mean ratio 1.9 ± 0.1 for *Gpr37l1^–/–^*, significantly different from 1, *p* = 0.002). This potentiation response was not different between the two genotypes (*p* = 0.16).

These results suggest that prosaposin‐mediated signalling via GPR37L1 in astrocytes prevents the potentiation of neuronal NMDAR‐mediated responses seen during repeated or prolonged activation of these receptors. This could occur by prosaposin suppressing the release of a molecule from astrocytes that normally generates this potentiation (although the obvious candidates, d‐serine and TNF‐α, have been ruled out) or by prosaposin evoking the release of a molecule from astrocytes that suppresses the potentiation. To ask whether this suppression of NMDAR potentiation could confer neuroprotection during ischemia, by reducing NMDAR‐mediated neurotoxicity (Rothman and Olney, 1995), we carried out in vitro ischemia experiments.

### 3.8 *Gpr37l1* expression and activation are neuroprotective in ischemia

**Figure 8:**
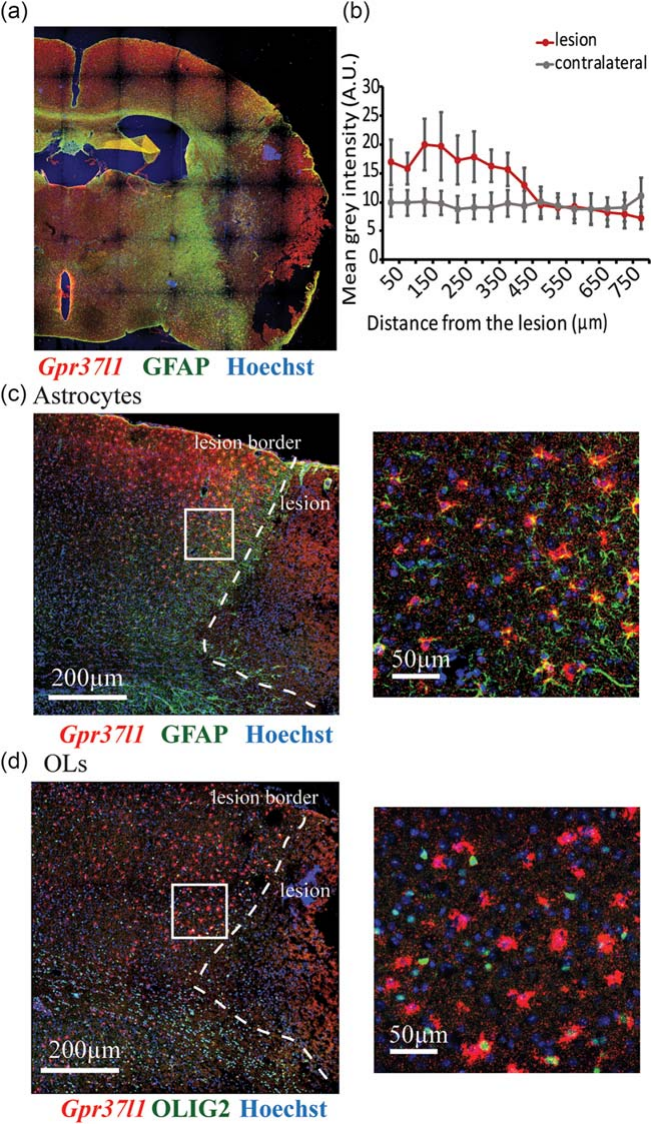
*Gpr37l1* expression is upregulated in astrocytes but not in OPs after MCAO. Expression of *Gpr37l1* 1 week after MCAO (30 min) in the lesioned hemisphere of mice was examined by ISH followed by immunolabeling. (a) MCAO induced a cortical lesion that is identifiable by the presence of necrotic tissue surrounded by a glial scar (b) The mean intensity of the *Gpr37l1* signal was quantified in a rectangle (800‐µm long, 500‐µm deep from the pial surface) starting at the edge of the lesion (as in a). Data are mean ± s.e.m of six experiments (one‐way ANOVA shows a significant decrease with distance in the lesioned hemisphere [*p* = 0.029] but not in the contralateral hemisphere [*p* = 0.9998]). (C, D) *Gpr37l1* was upregulated in cells at the lesion border. These *Gpr37l1^+^* cells in the penumbra were mostly GFAP‐positive (C) and OLIG2‐negative (D)

We examined the expression of *Gpr37l1* in GFAP‐positive astrocytes and in OLIG2‐positive OL‐lineage cells in the brain 7 days after 30 minutes of MCAO, by combining ISH with immunohistochemistry. *Gpr37l1* expression was significantly higher in cells immediately adjacent to the lesion area (Figure 8a), compared to the contralateral hemisphere, and decreased with distance from the lesion (Figure 8b, *n* = 6, one‐way ANOVA, *p* = 0.029 for the lesioned hemisphere, *p* = 0.9998 for the contralateral hemisphere). The cells that increased their expression of *Gpr37l1* were mainly GFAP‐positive astrocytes and not OPs (Figure 8c,d).

**Figure 9:**
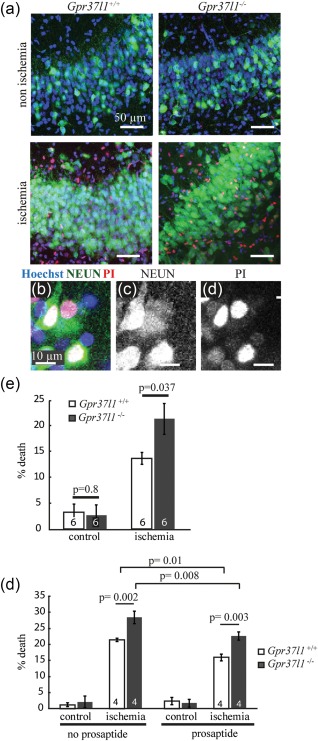
GPR37L1 is neuroprotective during chemical ischemia in vitro. Hippocampal slices from P14‐P16 *Gpr37l1* ^+/+^ and *Gpr37l1* ^–/–^ mice were incubated for 30 min in control or ischemic solution containing propidium iodide (PI), followed by 40 min in nonischemic solution, and subsequently labelled for NeuN. (a) NeuN cells labelled for PI were visible after ischemia in the pyramidal layer. (b) Example of a *Gpr37l1* ^+/+^ pyramidal neuron labelled for NeuN (c) and PI (d) after ischemia. (e) Percentage of dead cells for control or ischemia in *Gpr37l1* ^+/+^ and *Gpr37l1* ^–/–^ littermates (*n* = 6 experiments). (f) Percentage of dead cells for control or ischemia, alone or with prosaptide (pro) included in the ischemic solution, in *Gpr37l1* ^+/+^ and *Gpr37l1* ^–/–^ littermates (*n* = 4 experiments). All *p* values are corrected for multiple comparisons

Next, we assessed the function of GPR37L1 in hippocampal slices of P14‐P16 mice subjected to chemical ischemia for 30 min (see Methods). Cell death was minimal for both *Gpr37l1* ^+/+^ and the *Gpr37l1* ^–/–^ slices after 30 min in the control non‐ischemic solution (3.2% ± 1.6% of pyramidal layer neurons and 1.7% ± 0.8% of stratum radiatum astrocytes in the *Gpr37l1* ^+/+^, and 2.6% ± 2.0% of neurons and 2.3% ± 0.8% of astrocytes in the *Gpr37l1* ^–/–^, in 12 slices from 6 mice of each genotype). Cell death was detectable after 30 min of chemical ischemia, followed by 40 min in nonischemic (“reperfusion”) solution, in the CA1 pyramidal layer (where nuclear propidium iodide [PI] staining overlapped with the neuronal marker, NEUN; Figure 9a–d) and in the stratum radiatum (where PI staining was often surrounded by cytosolic GFAP staining, suggesting that many dead cells were astrocytes; Supporting Information Figure 5a–d). The presence of GPR37L1 significantly prevented ischemia‐evoked cell death in the pyramidal cell layer [Figure 9e, 13.6% ± 1.1% in *Gpr37l1* ^+/+^ slices compared to 21.3% ± 2.9% in *Gpr37l1* ^–/–^ slices, *n* = 6 (12 slices from 6 mice of each genotype), one‐way ANOVA *p* = 0.037]. There was, however, no difference in cell death for astrocyte somata in the stratum radiatum (16.0% ± 1.6% PI staining in *Gpr37l1* ^+/+^ compared to 17.3% ± 2.9% in *Gpr37l1* ^–/–^, *n* = 6 [12 slices from 6 mice of each genotype], one‐way ANOVA *p* = 0.7, data not shown). Thus, GPR37L1 is neuroprotective in the ischemic hippocampus.

The addition of prosaptide to boost GPR37L1 signaling significantly reduced cell death in the pyramidal layer of the hippocampus in *Gpr37l1* ^+/+^ slices (a ∼25% decrease comparing cell death in ischemia with or without prosaptide, *n* = 4, *p* = 0.01, Figure 9f). Prosaptide also decreased cell death in the hippocampus of *Gpr37l1* ^–/–^ mice (∼20% decrease, *n* = 4, *p* = 0.008, Figure 9f). However, prosaptide did not reduce ischemia‐evoked cell death in the stratum radiatum in either the *Gpr37l1* ^+/+^ or the *Gpr37l1* ^–/–^ slices (1.1% increase in death in *Gpr37l1* ^+/+^ slices, and 0.9% increase in *Gpr37l1* ^–/–^ slices, data not shown).

Thus, the activation of GPR37L1 that occurs in ischemia in the absence of added prosaptide (presumably caused by release of endogenous prosaposin) is neuroprotective for pyramidal neurons of the hippocampus and this protective effect is amplified when GPR37L1 is stimulated further by bath application of prosaptide. The fact that prosaptide is also neuroprotective in the *Gpr37l1* ^–/–^ mice suggests that the neuroprotective effect of prosaposin might also rely partly on other receptors such as GPR37 (expressed in mature OLs, Figure 3), or on other unknown mechanisms.

## 4 DISCUSSION

Prosaposin has been reported to be neuroprotective in ischemia, and glioprotective in conditions of oxidative stress (Lu, Otero, Hiraiwa, & O'Brien, 2000; Meyer et al., 2013; Morita et al., 2001; Sano et al., 1994; Terashita et al., 2013). Here we characterize one of the receptors that prosaposin acts through, GPR37L1.

We show that *Gpr37l1* is expressed in most or all astrocytes and a subset of OPs (Figure 1). The expression pattern differed from that of the related receptor *Gpr37*, which was mainly in mature OLs and not in astrocytes (Figure 3), contradicting a report that *Gpr37* is expressed in cultured astrocytes (Meyer et al., 2013) but consistent with transcriptome data (Zhang et al., 2014). In mice, *Gpr37l1* expression increases over the first postnatal month and continues to be expressed in adulthood (Figure 2) implying a role, not just in development, but in the function of the mature nervous system. Surprisingly, we could not verify an earlier claim that GPR37L1 deletion affects motor performance (Marazziti et al., 2013), possibly due to the use of different *Gpr37l1^‐/‐^* mouse lines with different genetic backgrounds, and we found no obvious effect on OP proliferation. *Gpr37l1* expression also had no effect on the resting electrical properties of hippocampal pyramidal neurons or astrocytes (Figure 4) but it had two potentially important effects on glutamatergic signalling.

First, although expression of *Gpr37l1* did not affect expression of the astrocyte glutamate transporters GLT‐1 and GLAST, activation of GPR37L1 with the prosaposin cleavage product prosaptide inhibited astrocyte glutamate uptake and this effect was abolished in the *Gpr37l1* KO (Figure 5). This suggests that prosaptide was acting through GPR37L1 receptors expressed on the astrocytes being recorded from, presumably [since GPR37L1 is coupled to G_i_ proteins; Meyer et al. (2013)] by lowering the cyclic AMP level in the astrocyte, altering phosphorylation by protein kinase A and thereby affecting the transporter cycling rate or trafficking of the transporter to and from the plasma membrane. Deleting *Gpr37l1* did not affect the uptake current in the absence of applied prosaptide (Figure 5), suggesting that there is normally little tonic release of prosaposin (at least in brain slices) and little spontaneous activity of the GPR37L1 receptor, contradicting the suggestion (Coleman et al., 2016) that GPR37L1 is spontaneously active (although we cannot rule out the possibility of compensation in response to the knock‐out). However, prosaposin expression and release are increased in ischemia (Costain et al., 2010; Hiraiwa et al., 2003) and we found that expression of *Gpr37l1* is increased in the penumbra of lesions caused by MCAO (Figure 8), so it is likely that glutamate transport activity is inhibited in these conditions.

If mild ischemia inhibits glutamate uptake, there is expected to be a rise in extracellular glutamate concentration, which might desensitize AMPARs and tonically activate NMDARs, thus altering neuronal information processing. A further suppression of glutamate transport by prosaposin release in this situation will accentuate these effects. The situation is different in profound ischemia, however, when ion gradient run‐down leads to glutamate transporters reversing and releasing glutamate, which reaches a concentration of 100–200 μM in the extracellular space and evokes a neurotoxic entry of Ca^2+^ via NMDAR channels (Krzyzanowska, Pomierny, Filip, & Pera, 2014; Rossi et al., 2000; Rothman & Olney, 1995). In this situation, inhibition of glutamate transport by prosaposin release will slow the release of glutamate. However, at least in the first few minutes of ischemia, transporter knock‐out experiments measuring the latency to the anoxic depolarization (when the extracellular glutamate concentration rises dramatically) suggest that it is the neuronal glutamate transporters that reverse first rather than astrocyte transporters, probably because the intracellular glutamate concentration is higher in neurons than in astrocytes (Gebhardt, Körner, & Heinemann, 2002; Hamann, Rossi, Marie, & Attwell, 2002).

Second, and perhaps more importantly, prosaptide‐evoked GPR37L1 signalling decreases the response of neurons to prolonged activation of NMDARs (Figure 6). Such prolonged activation will occur during the prolonged elevation of extracellular glutamate concentration that occurs in ischemia and GPR37L1 should thus decrease the neurotoxic rise of [Ca^2+^]_i_ that occurs in neurons in ischemia. Indeed, expression of GPR37L1 was neuroprotective during ischemia even in the absence of added prosaptide (Figure 9)—an effect that presumably depends on the release of prosaposin that is induced by ischemia (Costain et al., 2010; Hiraiwa et al., 2003; Yokota et al., 2001). In vivo, up‐regulation of *Gpr37l1* in the penumbra of an ischemic lesion (Figure 8) might promote GRP37L1‐mediated neuroprotection. The mechanism by which GPR37L1 decreases neuronal responses to prolonged activation of NMDARs is mysterious. Because the GPR37L1 is located in astrocytes, to regulate neuronal NMDARs a gliotransmitter of some sort must have its release from the astrocytes modulated when GPR37L1 is activated. We have ruled out two candidates for this role—d‐serine and TNF‐α —which have previously been shown to increase NMDAR responses when released from astrocytes (Henneberger et al., 2010; Shigetomi et al., 2013; Figure 7). Our work suggests that a further gliotransmitter must exist that has a similar effect, and that its release is modulated by GPR37L1, but further work is needed to identify this agent.

We found that *Gpr37l1* is also expressed in ∼25% of OPs but we did not detect any effect of *Gpr37l1* knockout on OP density or myelination in healthy mice (Supporting Information Figure 3b,c). However, GPR37 and GPR37L1 might protect against demyelination caused by injury or disease, and/or stimulate remyelination (Hiraiwa, Campana, Mizisin, Mohiuddin, & O'Brien, 1999; Hiraiwa, Taylor, Campana, Darin, & O'Brien, 1997). Myelinating OLs are sensitive to ischemia (Back & Rosenberg, 2014) and are probably damaged in our in vitro ischemia experiments, but we did not quantify this. The potential glioprotective role of GPR37L1 during ischemia or other insults, and what distinguishes the GPR37L1‐expressing and nonexpressing subpopulations of OPs, are interesting questions for the future.

## Supporting information

Additional Supporting Information may be found online in the supporting information tab for this article.

Supporting Information Figure 1

Supporting Information Figure 2

Supporting Information Figure 3

Supporting Information Figure 4

Supporting Information Figure 5

Supporting Information Figure 6

Supporting Information Figure 7
