# Supplementary figures and images for: G protein‐coupled receptor 37‐like 1 modulates astrocyte glutamate transporters and neuronal NMDA receptors and is neuroprotective in ischemia
Source: Glia. 2017 Aug 10;66(1):47–61. doi: 10.1002/glia.23198 (PMC5724489; doi:10.1002/glia.23198)

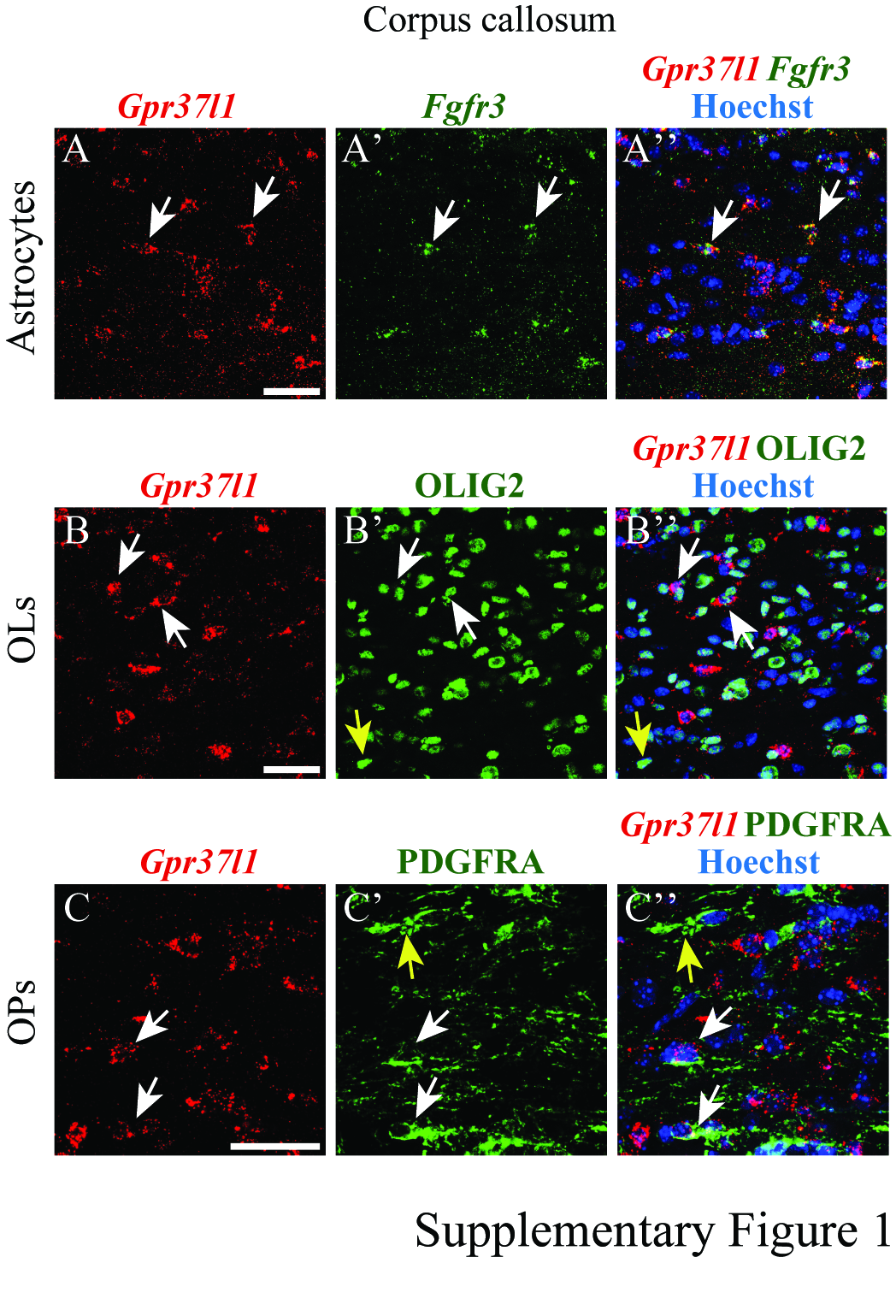

Supplement: Supplementary file 1 — Supporting Information Figure 1 [file GLIA-66-47-s001.tif]

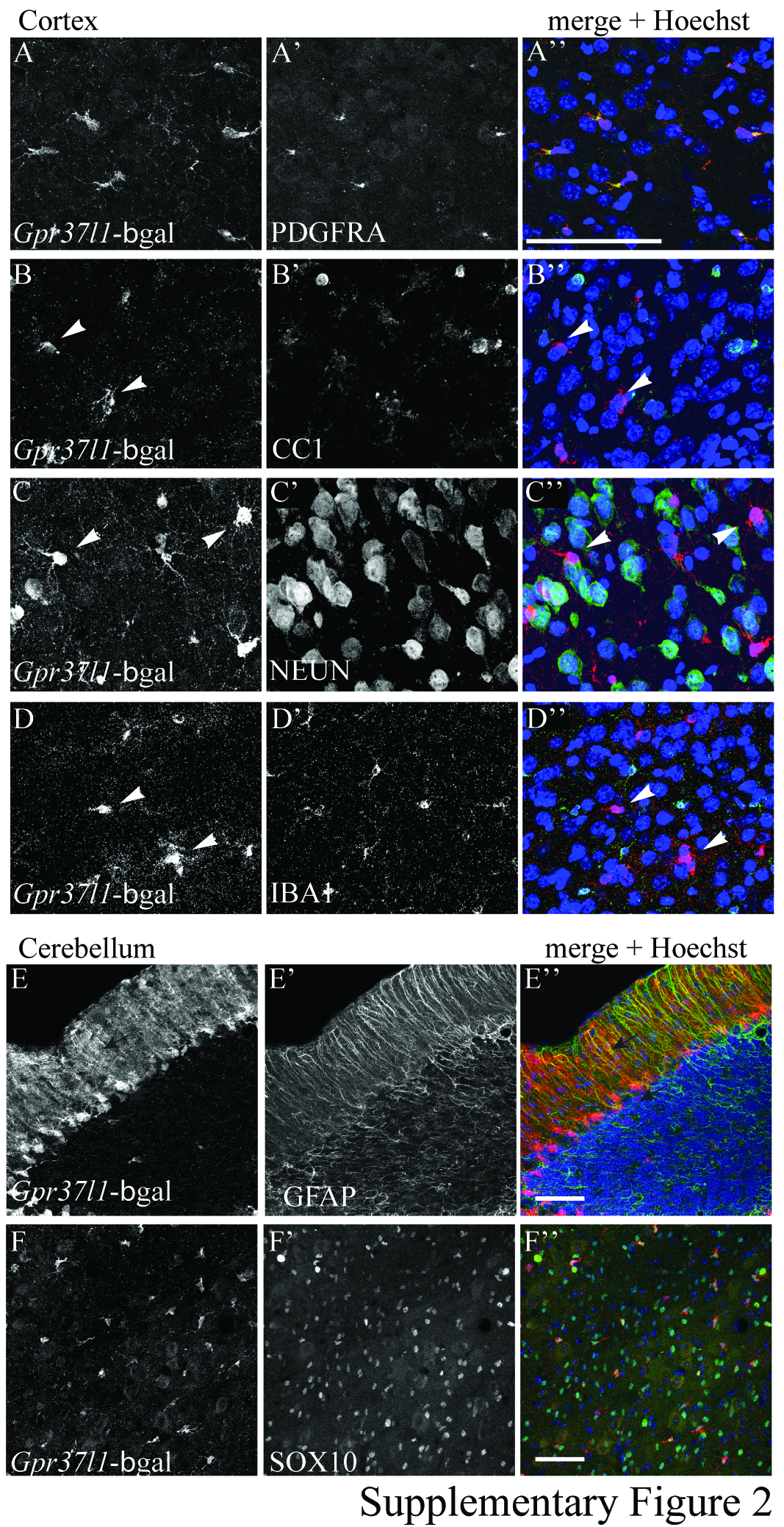

Supplement: Supplementary file 2 — Supporting Information Figure 2 [file GLIA-66-47-s002.tif]

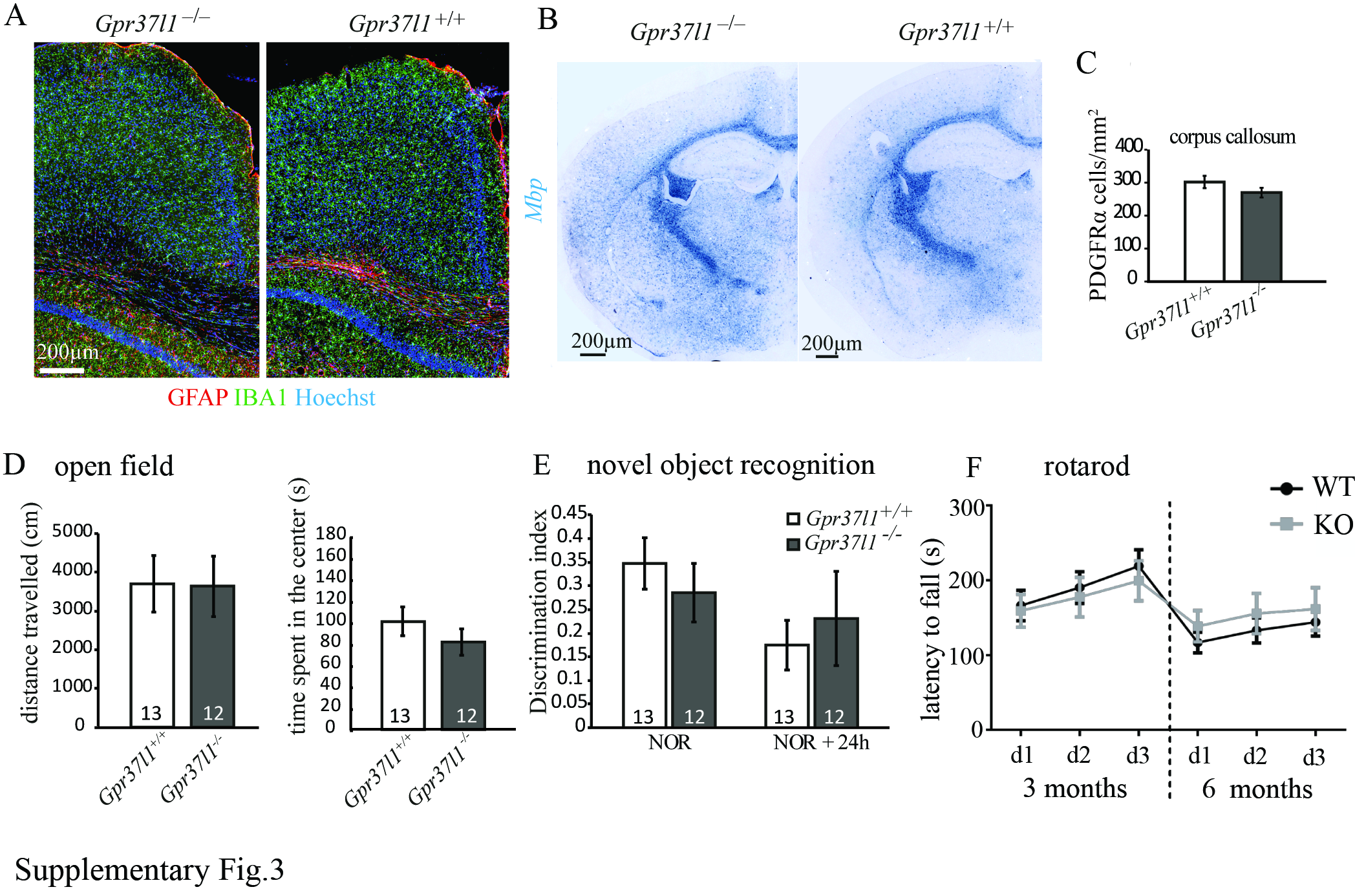

Supplement: Supplementary file 3 — Supporting Information Figure 3 [file GLIA-66-47-s003.tif]

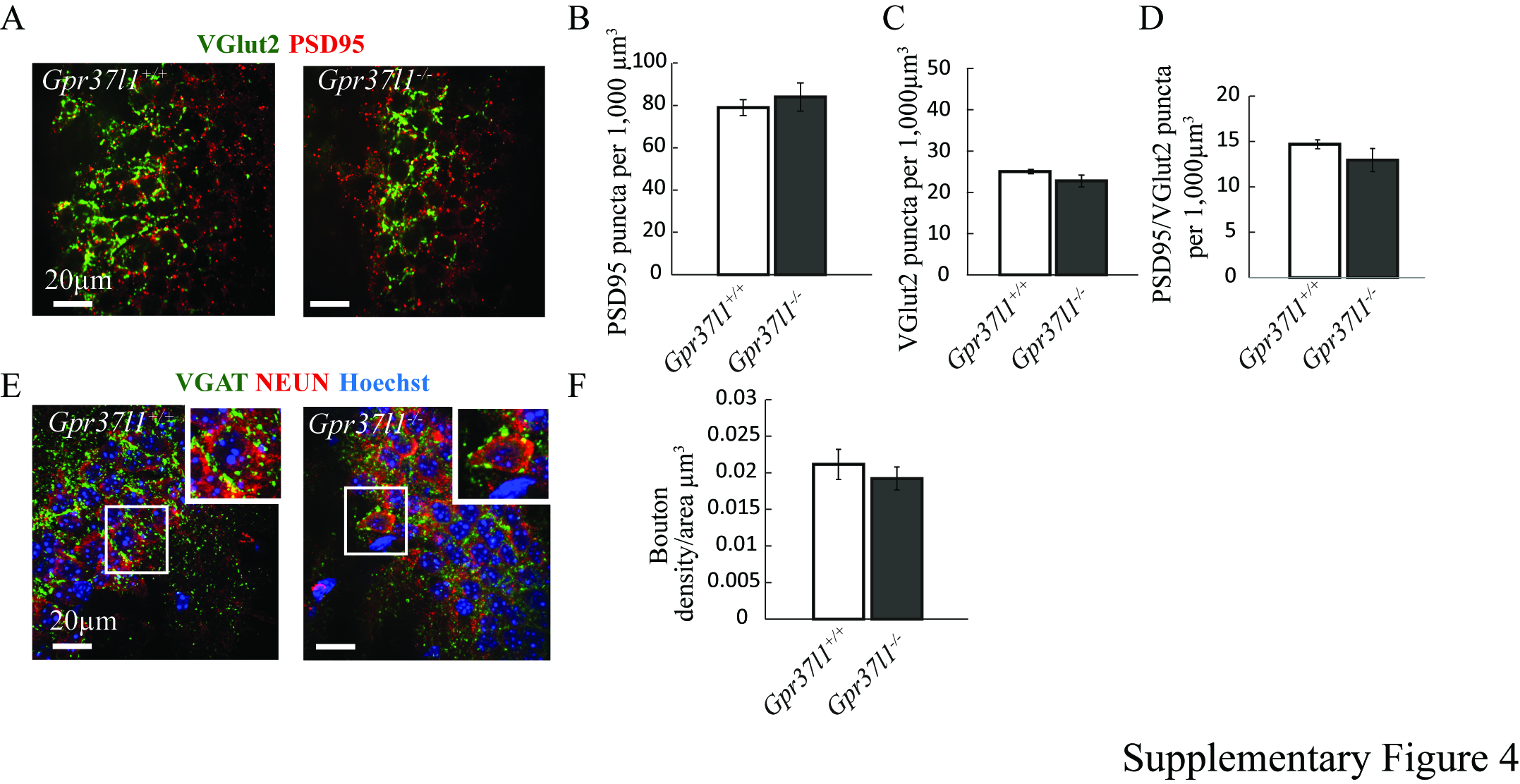

Supplement: Supplementary file 4 — Supporting Information Figure 4 [file GLIA-66-47-s004.tif]

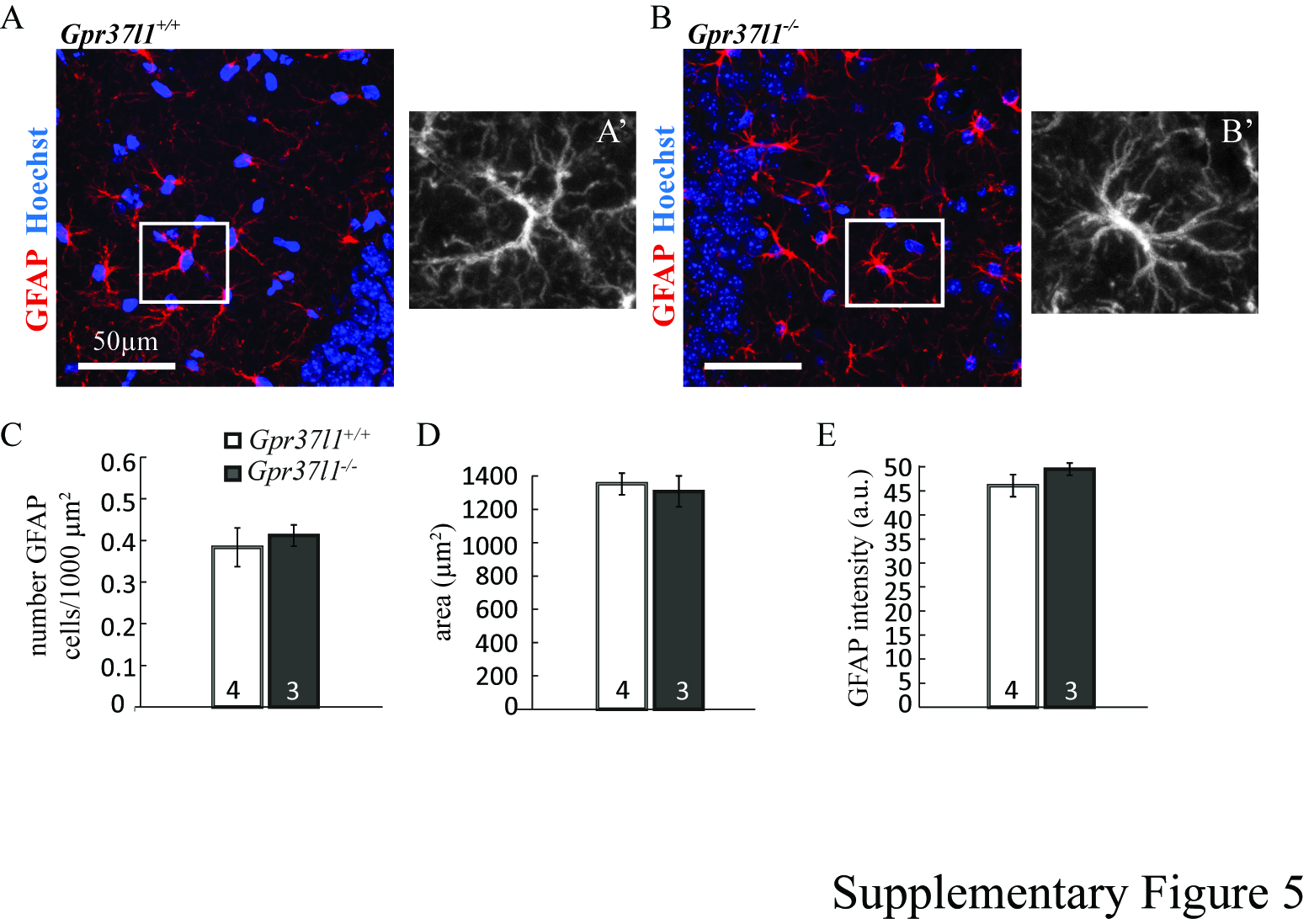

Supplement: Supplementary file 5 — Supporting Information Figure 5 [file GLIA-66-47-s005.tif]

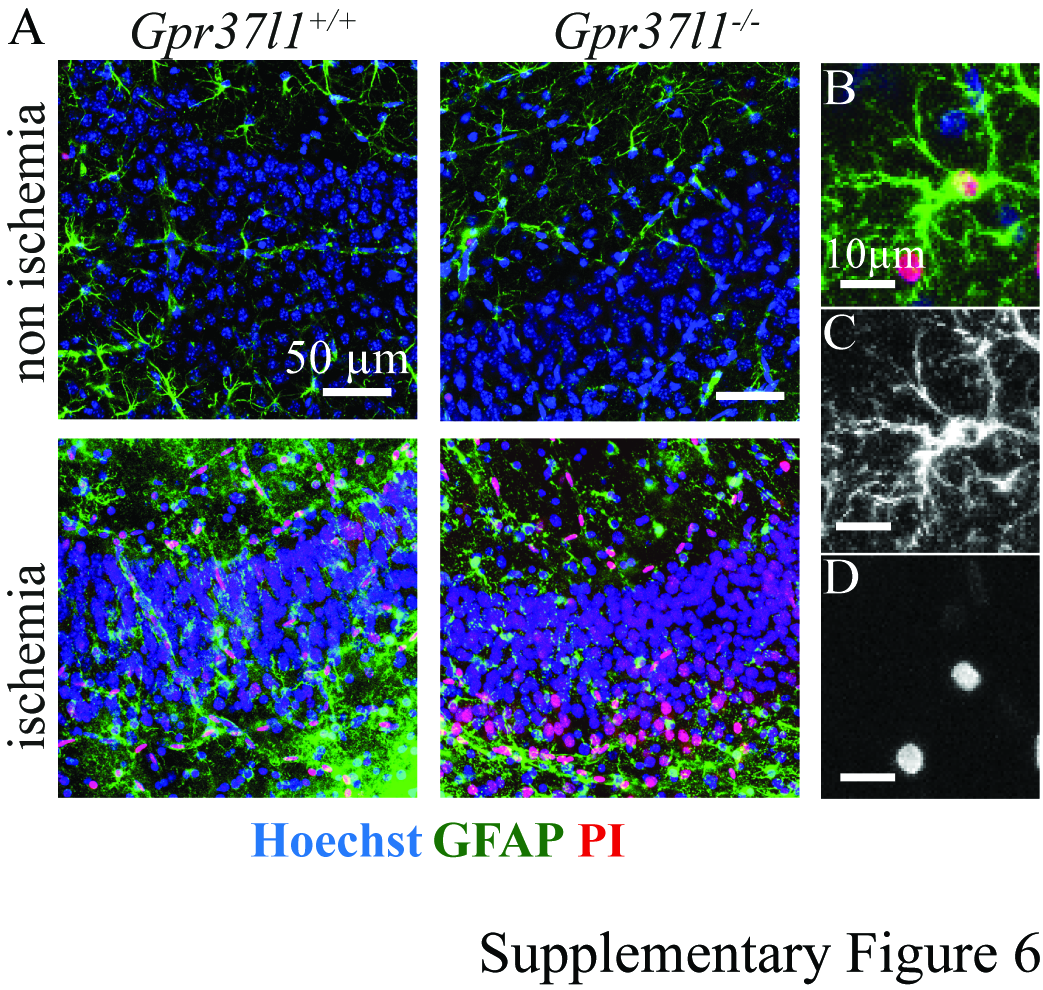

Supplement: Supplementary file 6 — Supporting Information Figure 6 [file GLIA-66-47-s006.tif]

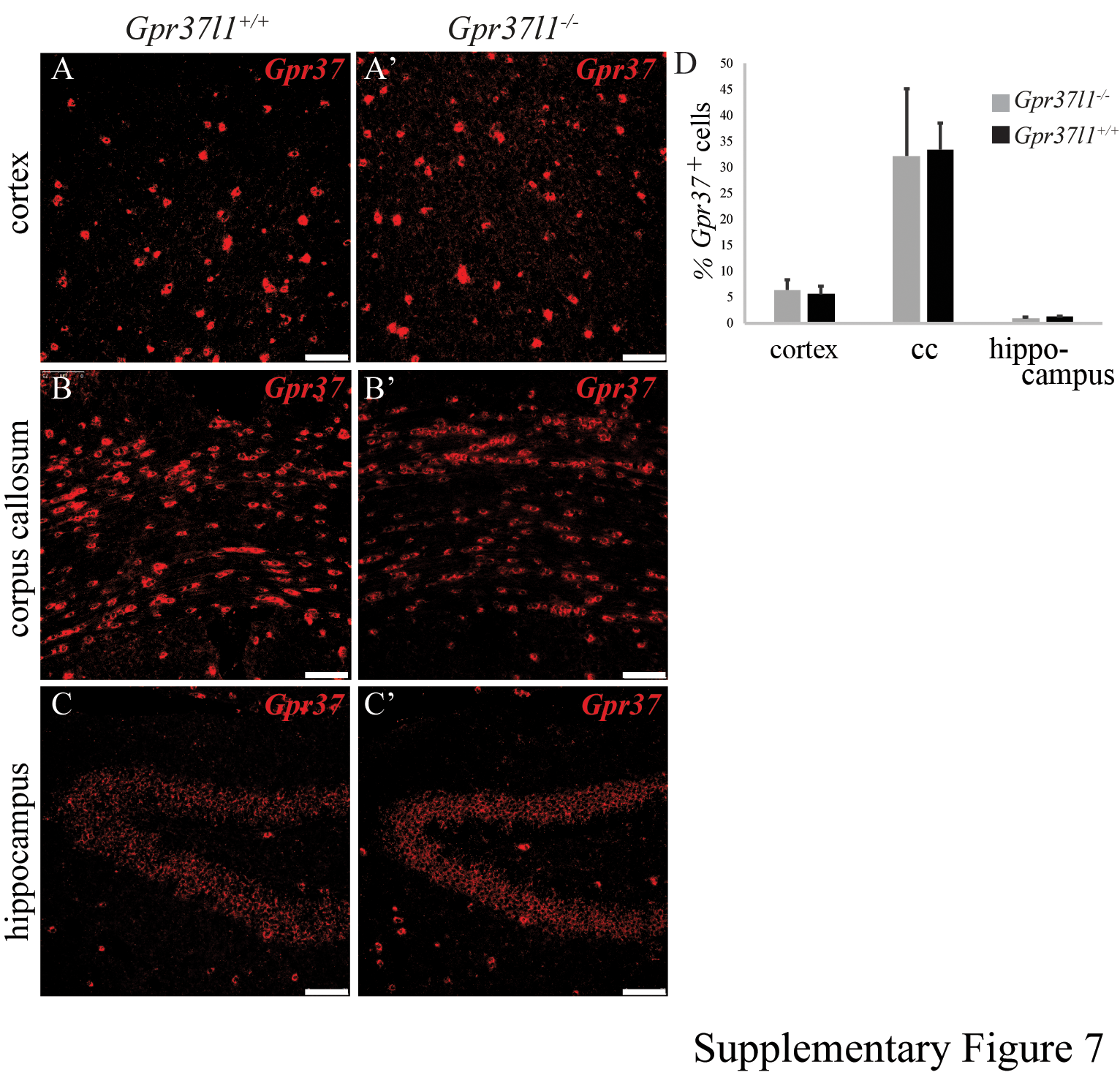

Supplement: Supplementary file 7 — Supporting Information Figure 7 [file GLIA-66-47-s007.tif]
